# Cyclin-Specific Docking Mechanisms Reveal the Complexity of M-CDK Function in the Cell Cycle

**DOI:** 10.1016/j.molcel.2019.04.026

**Published:** 2019-07-11

**Authors:** Mihkel Örd, Rainis Venta, Kaidi Möll, Ervin Valk, Mart Loog

**Affiliations:** 1 Institute of Technology, University of Tartu, Tartu 50411, Estonia

**Keywords:** cyclin-dependent kinase, mitosis, CDK, kinase specificity, cell cycle, *Saccharomyces cerevisiae*, kinase docking, CDK inhibition

## Abstract

Cyclin-dependent kinases (CDKs) coordinate hundreds of molecular events during the cell cycle. Multiple cyclins are involved, but the global role of cyclin-specific phosphorylation has remained unsolved. We uncovered a cyclin docking motif, LxF, that mediates binding of replication factor Cdc6 to mitotic cyclin. This interaction leads to phospho-adaptor Cks1-mediated inhibition of M-CDK to facilitate Cdc6 accumulation and sequestration in mitosis. The LxF motif and Cks1 also mediate the mutual inhibition between M-CDK and the tyrosine kinase Swe1. Additionally, the LxF motif is critical for targeting M-CDK to phosphorylate several mitotic regulators; for example, Spo12 is targeted via LxF to release the phosphatase Cdc14. The results complete the full set of G1, S, and M-CDK docking mechanisms and outline the unified role of cyclin specificity and CDK activity thresholds. Cooperation of cyclin and Cks1 docking creates a variety of CDK thresholds and switching orders, including combinations of last in, first out (LIFO) and first in, first out (FIFO) ordering.

## Highlights

• Mitotic cyclin Clb2 binds a specific linear motif, LxF, in targets or inhibitors
• LxF interaction enhances mitotic CDK substrate phosphorylation
• Phospho-adaptor Cks1 and the LxF docking mediate CDK inhibition by Cdc6 and Swe1
• Cyclin-specific targeting enables finetuning of CDK function

## Graphical Abstract

**Figure:**
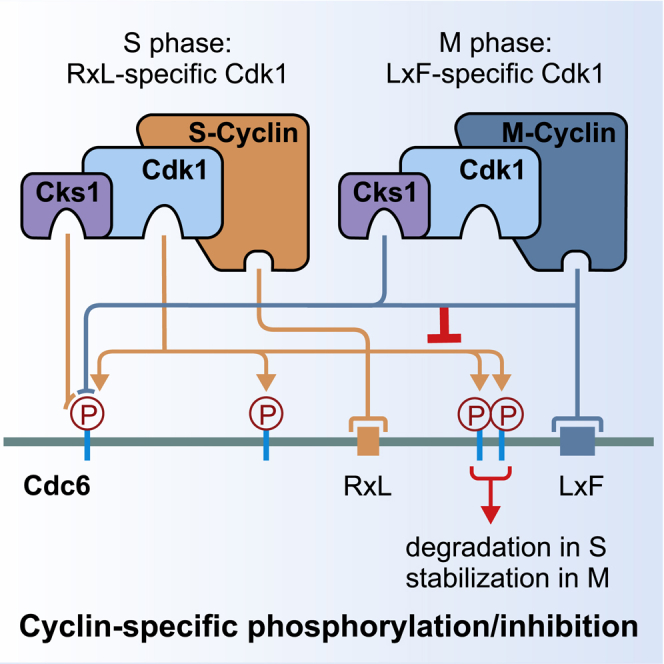

## Teaser

Örd et al. find that a short linear motif in proteins enables specific targeting by the mitotic CDK. Depending on the context, this motif can lead to either enhanced phosphorylation of mitotic targets or inhibition of the CDK complex in cooperation with the phospho-adaptor subunit Cks1.

## Introduction

To accomplish one of the most complex biological tasks, the assembly of an extra copy of the cell, cyclin-dependent kinases (CDKs) coordinate hundreds of events during the cell cycle (Morgan, 2007). In this process, CDKs catalyze thousands of phosphorylation events. It has been estimated that CDK in budding yeast has about 500–700 targets (∼10% of all genes), and the phospho-regulation of close to 100 of these has been functionally characterized (Ubersax et al., 2003, Enserink and Kolodner, 2010).

Although it is well established that the cell cycle is coordinated by CDK, it has been difficult to outline a systems-level mechanism for the temporal organization of CDK-driven events. Besides a very coarse-grained categorization of early, mid, and late targets (Swaffer et al., 2016), cyclin specificities (Schulman et al., 1998, Wilmes et al., 2004, Loog and Morgan, 2005, Bhaduri and Pryciak, 2011, Kõivomägi et al., 2011b), and phosphatase dynamics (Queralt et al., 2006, De Wulf et al., 2009, Godfrey et al., 2017), we currently have quite a blurred picture of phosphorylation order, CDK thresholds, and changes in CDK substrate specificities during the cycle. According to the threshold model, accumulating CDK activity triggers all major cell cycle events at different thresholds (Stern and Nurse, 1996, Coudreuse and Nurse, 2010, Swaffer et al., 2016). The threshold model was derived from elegant work on fission yeast, where doses of CDK inhibitor were used to determine CDK activity thresholds for S phase and mitotic transitions (Coudreuse and Nurse, 2010), and it has served as a basic framework for CDK function.

In addition, experimental evidence suggests that cyclin-specific substrate recognition also controls the temporal order of CDK-driven switches. Cyclins affect CDK specificity in two ways. First, it was found in yeast and then in mammals (Topacio et al., 2019) that cyclins modulate the active site specificity of CDK in such a way that the activity toward a substrate peptide increases in the order that cyclins appear in the cell cycle (Loog and Morgan, 2005, Kõivomägi et al., 2011b). Second, although G1- and S-CDK have lower active site activity, they bind linear docking motifs in substrates using docking pockets on cyclins to increase their specificity toward a set of targets (Schulman et al., 1998, Wilmes et al., 2004, Loog and Morgan, 2005, Bhaduri and Pryciak, 2011, Kõivomägi et al., 2011b, Kõivomägi and Skotheim, 2014, Örd and Loog, 2019). Budding yeast G1-CDK uses a substrate LP (leucine- and proline-rich) motif and the S-CDK an RxL motif to mediate interactions with specific substrates (Loog and Morgan, 2005, Bhaduri and Pryciak, 2011, Kõivomägi et al., 2011b).

The mitotic cyclins have several specific functions. In budding yeast, the mitotic cyclins Clb2 and Clb1 initiate anaphase and promote the isotropic growth switch in early mitosis and spindle elongation during anaphase (Dahmann and Futcher, 1995, Eluère et al., 2007, Rahal and Amon, 2008, Machu et al., 2014). Additionally, only mitotic Cdk1 complexes are subjected to inhibitory phosphorylation by Swe1 (Hu and Aparicio, 2005, Keaton et al., 2007). Also, degradation of Clb2 (mitotic CDK [M-CDK]) is essential for mitotic exit whereas degradation of Clb5 (S-CDK) and Clb3 (G2-CDK) is not, indicating that only M-CDK inhibits mitotic exit (Wäsch and Cross, 2002, Pecani and Cross, 2016). Until now, there has been no reported evidence of specific docking mechanisms for M-CDK. Because M-CDK coordinates the most crucial stage of cell division, this missing part of the puzzle has been an obstacle to understanding all aspects of CDK function in the cell cycle.

**Figure 1:**
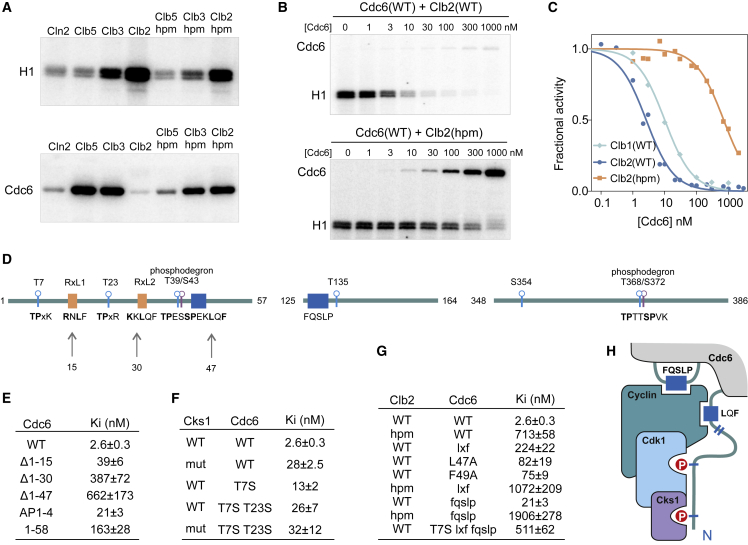
Cdc6 Inhibits M-CDK via Cks1 and an LxF Motif (A) G1-, S-, and M-Cdk1 complexes show different specificity toward Cdc6. Equal concentrations of four representative Cdk1 complexes and versions with mutated cyclin docking sites (*hpm*) were used in a kinase assay with recombinant Cdc6. Histone H1 was used as a control substrate. Autoradiographs of SDS-PAGE are shown. (B) To test the inhibition of M-CDK by Cdc6, increasing amounts of purified Cdc6 were used in a histone H1 kinase assay with either wild-type M-CDK or its *hpm* version. (C) Quantified inhibition profiles obtained from assays using histone H1 and Cdc6. (D) Diagrams showing CDK phosphorylation motifs, degrons, and potential cyclin docking motifs in key disordered regions of Cdc6. (E–G) The K_i_ values for inhibition of M-CDK histone H1 phosphorylation activity by Cdc6 and its various mutants were measured with either wild-type Cks1 or Cks1(mut) (phosphopocket mutant, F). (E) The effect of truncation of Cdc6 and mutation of phosphorylation sites on the K_i_ values. Cdc6(AP1–4) carries mutations T7A T23A T39A S43A. (F) The effect of Cks1 on inhibition. (G) Mapping of additional inhibitory interactions. Cdc6(lxf) carries L47A F49A, and Cdc6(fqslp) carries the triple mutation F126A L129A P130A. (H) Scheme showing the inhibition mechanism of M-CDK with Cdc6 as a phospho-primed inhibitor. See also Figure S1.

Previously, we found an example of extreme cyclin specificity that sharply discriminates between the closely related B-type cyclins of budding yeast, the Clbs (Kõivomägi et al., 2011b). Cdc6, a key protein that controls replication origin licensing, was found to be a specific target for Clb5- and Clb3-Cdk1 but displayed a very poor phosphorylation rate with the mitotic Clb2-Cdk1 (Figure 1A). Paradoxically, pull-down assays showed Cdc6 binding specificity for Clb2 but not for Clb5 or Clb3 (Archambault et al., 2003, Mimura et al., 2004).

Phosphorylation of Cdc6 determines the time window for replication origin licensing (Calzada et al., 2000, Calzada et al., 2001). Phosphorylation of Cdc6 by CDK directs it to degradation via the Skp, Cullin, F-box (SCF)-proteasome system (Drury et al., 2000). Low CDK activity in G1 allows the origin recognition complex (ORC) and Cdc6 to load the Mcm helicase at the origins. At G1-S, DNA replication is triggered by phosphorylation of Sld2 and Sld3 by S-CDK (Tanaka et al., 2007, Zegerman and Diffley, 2007). In parallel, ORC, Cdc6, and the Mcm complex are phosphorylated by S-CDK to prevent origin re-licensing and re-replication (Nguyen et al., 2001, Wilmes et al., 2004). As a secondary function of Cdc6 phosphorylation, it has been proposed that M-CDK binds to the phosphorylated N-terminal domain of Cdc6, forming a tight complex that inhibits the origin licensing function of Cdc6 (Mimura et al., 2004). Also, Cdc6 has been proposed to cooperate with Sic1 and Cdh1 during mitotic exit to suppress M-CDK activity by inhibition (Calzada et al., 2001). Archambault et al. (2003) have argued against this idea by showing that although Cdc6 has the potential to inhibit M-CDK, it is not required for mitotic exit to occur.

In the present study, we dissected the mechanism of Cdc6-mediated inhibition and present a unique inhibitory mechanism that involves an M-CDK-specific cyclin docking motif, LxF, in Cdc6 and the phospho-adaptor Cks1, that leads to shielding of the degron and sequestration of Cdc6 by M-CDK during mitotic exit. In addition, the M-CDK docking motif was found to play a broader role in CDK function during mitosis. The docking motif is essential for phosphorylation of Spo12 and activation of the fourteen early anaphase release (FEAR) network; it targets M-CDK activity during the isotropic growth switch, directs Clb2 localization to the bud neck, and enables specific regulation of M-CDK by Swe1. Finally, we show that the CDK threshold and the cyclin specificity models are not alternatives; instead, our data lead to a unified model of CDK function according to which cyclin specificity and Cks1 mechanisms provide flexibility for creating many CDK thresholds and complex temporal switching orders.

## Results

### Cdc6 Inhibits Mitotic CDK, but Not G1-, S-, or G2-CDK

Using purified Cdc6 and four cyclin-Cdk1 complexes, we found that although it is a poor substrate for Cln2 (G1-CDK) and Clb2 (M-CDK) complexes, Cdc6 was efficiently phosphorylated by both Clb5 (S-CDK) and Clb3 (G2-CDK) complexes (Figure 1A). Phosphorylation by S-CDK was dependent on a known substrate docking pocket of cyclins, the hydrophobic patch (*hp*), and mutation of the pocket (hydrophobic patch mutant [*hpm*]) reduced the phosphorylation rate. In contrast, a mutation in the *hp* of M-cyclin enhanced the phosphorylation of Cdc6. Such a remarkable cyclin-specific phosphorylation profile suggested that the interaction between Cdc6 and M-CDK could be inhibitory, whereas the S-CDK *hp* interaction promotes phosphorylation. Indeed, M-CDK was inhibited by Cdc6 with low nanomolar K_i_ values, whereas inhibition was weakened by more than two orders of magnitude in the *hpm* version (Figures 1B and 1C; Figure S1A).

### The Cdc6 Inhibitory Mechanism with Priming Phosphorylation for Cks1 Binding Is Mediated by an M-Cyclin-Binding LxF Motif

To map the critical elements in Cdc6 responsible for inhibition, we first analyzed the disordered N terminus (Figure 1D). Deletion of amino acids upstream of positions 15, 30, and 47 as well as mutation of the 4 N-terminal CDK sites reduced the inhibitory potency (Figure 1E; Figures S1A and S1B). These results agree with a previous report showing that co-precipitation of Clb2 and Cdc6 is dependent on the N-terminal phosphorylation sites (Mimura et al., 2004). The role of CDK sites in inhibition raised the possibility that the phosphorylated sites could bind to Cks1, the phospho-adaptor of the CDK complex. Mutation of the phosphate binding pocket of Cks1 (Cks1mut) reduced inhibition by about 10 times, much like the phosphorylation site mutants (Figure 1F; Figures S1A and S1C). Because Cks1 only binds phospho-threonines and not phospho-serines (Kõivomägi et al., 2013, McGrath et al., 2013), we mutated the N-terminal TP sites to SPs and also observed a loss of inhibition. Because the purified Cdc6 does not contain phosphorylated sites, M-CDK must phosphorylate the N-terminal sites upon formation of the inhibitory complex, which suggests a phospho-primed inhibition mechanism mediated by Cks1. Therefore, Cdc6 functions as a substrate that is phosphorylated by M-CDK and bound with nanomolar affinity, leading to inhibition. A quite analogous mechanism has been reported in PP2A-B55 inhibition by endosulfine, where the mechanism is called inhibition by unfair competition (Williams et al., 2014).

Because deletion of the N-terminal tail (Cdc6(Δ1–47)) reduced inhibition by more than 100-fold, whereas mutations in Cks1 and phosphorylation sites caused only a 10-fold reduction, we reasoned that the remaining affinity depends on a docking motif that binds to the *hp* of Clb2. Judging from the known distance requirements between the Cks1 binding sites, the active site, and the *hp* (Kõivomägi et al., 2013), we identified a conserved motif, ^47^LxF^49^, that is crucial for inhibition (Figure 1G; Figures S1A, S1D, and S4B). However, the N-terminal tail harboring the CDK sites and the LxF motif was not sufficient for low-nanomolar inhibition (Cdc6(1–58); Figure 1E). Additionally, a hydrophobic stretch (at 126–130) within a disordered loop of Cdc6 also contributed to inhibition (Figure 1G). The effect of LxF mutation (L47A F49A) showed very little additive effect when Clb2(*hpm*) was used instead of Clb2(wild-type [WT]). On the other hand, the mutation ^126^FQSLP^130^ reduces inhibition, even in the case of Clb2(*hpm*), suggesting that LxF binds to the *hp* (Figures 1F and 1G; Figure S1E). Therefore, we propose a mechanism where Cdk1 first phosphorylates the N-terminal TP sites, which then bind to Cks1, whereas LxF binds to the *hp* of Clb2, and, additionally, an interaction between ^126^FQSLP^130^ and M-CDK enhances the inhibition (Figure 1H).

### The Dynamics of Clb2 and Cdc6 Expression Reveal a Dual Role of Inhibition

**Figure 2:**
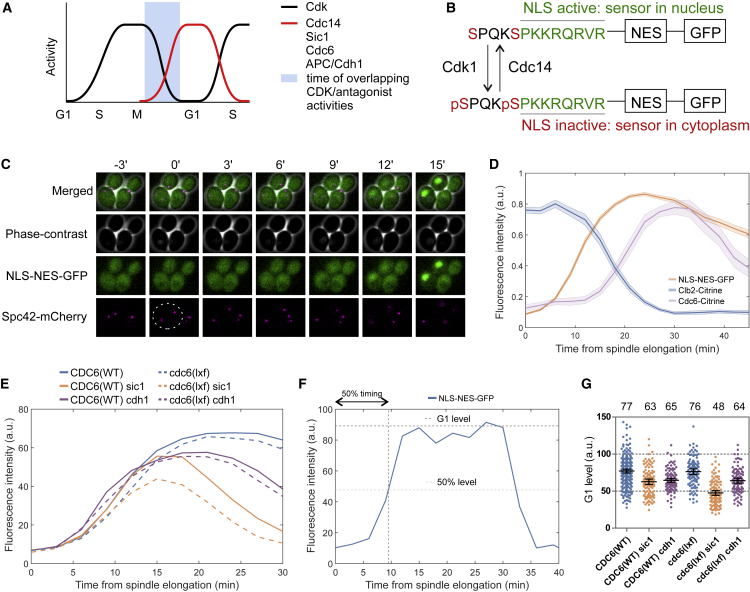
The *In Vivo* Dynamics of Clb2 and Cdc6 Expression Reveal a Dual Role of LxF (A) A scheme depicting the window between mitosis and G1 when opposite signals, CDK and its antagonists, including Cdc6, are simultaneously present. The red line represents the net effect profile of CDK antagonists to be measured by an NLS-NES Cdk1 activity sensor (see B). (B) Scheme explaining phospho-regulatable NLS-NES Cdk1 activity sensor. Phosphorylation of the SP sites leads to nuclear export of the sensor. (C) Time-lapse microscopy images showing cycling cells with the GFP-tagged Cdk1 activity sensor (NLS-NES-GFP) and mCherry-tagged SPBs (Spc42-mCherry). A white oval marks the time of spindle elongation. (D) Averaged normalized nuclear fluorescence intensities of NLS-NES-GFP, Clb2-Citrine, and Cdc6-Citrine as measured from spindle elongation. Error bars show ± SEM. (E) The effect of Sic1, Cdh1, and the *lxf* mutation in Cdc6 on the nuclear levels of the Cdk1 activity sensor from anaphase onset. The plots show the nuclear levels of the sensor averaged over a population of cells. (F) A scheme explaining the parameters of Cdk1 activity sensor nuclear accumulation (50% timing and G1 level) used in single-cell analysis. The G1 level was calculated as the average of the 3 highest intensities. (G) Plot showing the G1 nuclear level of the NLS-NES-GFP sensor in the indicated strains. The numbers above the plot show the average for each strain, and error bars indicate 95% confidence intervals. See also Figure S2.

The tight interaction between M-CDK and Cdc6 could have two roles: to inhibit CDK activity during mitotic exit and to prevent Cdc6 from licensing replication origins while mitotic cyclins are present (Figure 2A; Calzada et al., 2001, Archambault et al., 2003, Mimura et al., 2004). We set up a microscopy assay to follow the dynamics of Cdk1 activity and the levels of Clb2 and Cdc6 during the cell cycle. We used a phospho-regulated nulear localization signal (NLS) as a sensor for Cdk1 activity (NLS-nuclear export signal (NES)-GFP; Liku et al., 2005; Figure 2B) and anaphase onset as a reference point by following spindle elongation with Spc42-mCherry-labeled spindle pole body (SPB) (Figure 2C). To set the temporal framework, we first followed the levels of Clb2 and Cdc6 after onset of anaphase using strains with *CLB2-Citrine* and *CDC6-Citrine* (Figure 2D; Figures S2A and S2B). The peak of Clb2-Citrine was reached at about the point of spindle elongation, as averaged over a large sample of cells, whereas nuclear accumulation of the Cdk1 activity sensor as well as Cdc6 levels started to increase slightly after the Clb2 peak and reached their G1 maxima at about the 20- and 30-min time points, respectively. Therefore, the activation of phosphatase Cdc14 and dephosphorylation of CDK substrates starts at a time when Clb2 levels are still high, creating a situation where replication origin licensing and firing could occur simultaneously (Figure 2A). When *CDC6* was replaced by *cdc6(lxf)*, which contains the mutated docking motif, the Cdk1 activity sensor showed only a 1-min delay in nuclear accumulation (Figures 2E and 2F; Figure S2C). However, when *SIC1* was deleted in the background, the effect of the LxF mutation on the magnitude of nuclear accumulation of the sensor was prominent. This suggests that, in the absence of Sic1, Cdc6-mediated inhibition is essential for proper suppression of Cdk1 activity in G1 (Figures 2E–2G). In addition to Sic1 and Cdc6, a third negative regulator of Cdk1 in mitotic exit is the anaphase-promoting complex (APC), which is responsible for the destruction of cyclins (Visintin et al., 1997, Calzada et al., 2001, Archambault et al., 2003). Deletion of *CDH1*, the late mitotic activator of APC, caused a minor drop in G1 levels of the sensor, and the *cdc6(lxf)* mutation did not lead to a further decrease. These results agree with previous reports suggesting that Cdc6, Sic1, and Cdh1 cooperatively suppress M-CDK in mitotic exit (Calzada et al., 2001) but that Cdc6 does not play an essential part when the other factors are present (Archambault et al., 2003).

**Figure 3:**
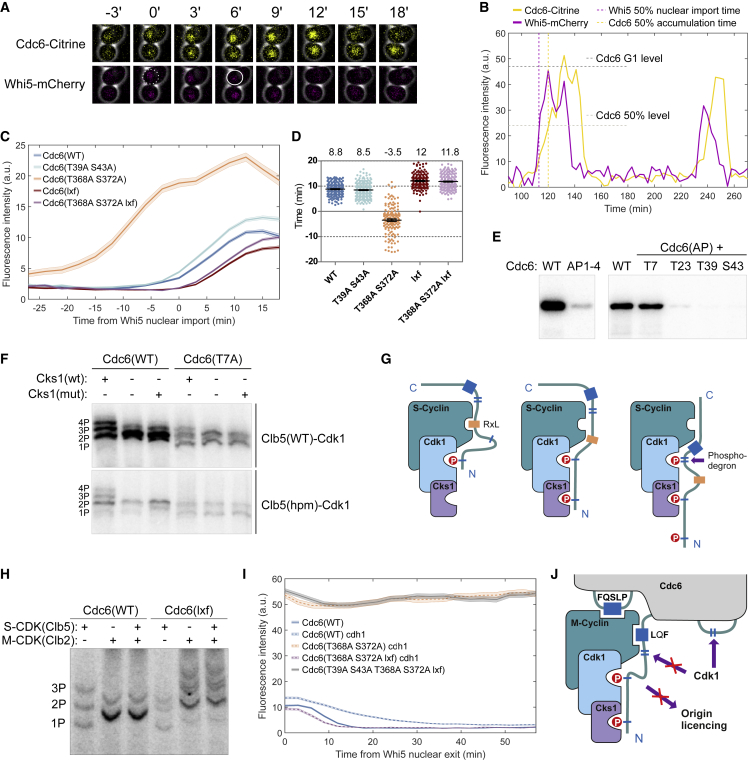
The N-Terminal Degron of Cdc6 Is Shielded by M-CDK (A) Time-lapse microscopy images of cells expressing Whi5-mCherry and Cdc6-Citrine. The dashed circle marks Whi5 nuclear entry in mitotic exit, and the solid circle shows Whi5 nuclear exit in late G1. (B) Quantified fluorescence profiles of a single cell from the experiment described in (A). (C) Average nuclear levels of Cdc6-Citrine mutants in mitotic exit. Time 0 denotes Whi5-mCherry nuclear levels reaching 50% of the G1 level. Error bars are ± SEM. (D) The time from nuclear import of 50% Whi5-mCherry to Cdc6 levels reaching 50% of their G1 level for the indicated Cdc6 mutants in single cells. The numbers above the plot show the average time. Error bars are 95% confidence intervals of the mean. (E) Autoradiograph showing *in vitro* phosphorylation of Cdc6 by S-CDK (Clb5). Cdc6(AP1-4) contains mutations T7A T23A T39A S43A, and in Cdc6(AP), all CDK consensus motifs except the indicated one were mutated. (F) The effect of *hp* of Clb5 and Cks1 on the phosphorylation of Cdc6(WT) or Cdc6(T7A). Autoradiographs of Phos-tag SDS-PAGE. (G) A scheme illustrating the docking and sequential dynamics of phosphorylation of the N-terminal tail of Cdc6 by S-CDK. (H) Shielding of the Cdc6 N-terminal degron by M-CDK in a kinase assay. Autoradiographs of a Phos-tag SDS-PAGE show multisite phosphorylation of Cdc6. Clb2-Cdk1 and Cdc6 were mixed in a 1:1 molar ratio. (I) Clb2 inhibits Cdc6 degradation during G1-S in *cdh1Δ* cells. Cdc6-Citrine levels were monitored in time-lapse microscopy experiments. The average Cdc6-Citrine fluorescence intensities of a population of cells after nuclear export of Whi5-mCherry are shown. (J) Diagram illustrating the shielding mechanism of the N-terminal degron and Cdc6 licensing activity in the Cdc6-M-CDK inhibitory complex. See also Figure S3.

In addition to the inhibitory function, phospho-regulation of Cdc6 controls its expression profile via phosphorylation of degrons (Calzada et al., 2000, Drury et al., 2000, Perkins et al., 2001). We analyzed the effect of mutations in the two SCF-binding degrons on Cdc6-Citrine levels (Figures 3A–3D; Figure S3A). Mutation of the N-terminal degron (T39A S43A) only moderately affected the accumulation of Cdc6 in mitotic exit, whereas mutation in the second degron (T368A S372A) had a profound effect, which suggests that degradation via the second degron controls Cdc6 levels in mitosis (Figures 3A–3D). Because the LxF motif partially overlaps with the N-terminal degron, we wondered whether the interaction with M-CDK may shield the degron from phosphorylation. Mutation of LxF in Cdc6 caused a modest decrease in Cdc6 levels, whereas addition of the LxF mutation to Cdc6(T368A S372A) reversed the effect of T368A S372A (Figures 3A–3D). These results support the hypothesis that the M-CDK *hp*-LxF interaction shields the N-terminal degron and directs the degradation of Cdc6 in mitosis mostly via the C-terminal degron.

Kinase assays performed with S-CDK suggest that mainly the N-terminal cluster in Cdc6 is targeted and that only T7 is a primary target (Figure 3E). However, phosphorylated T7 serves as a priming site for Cks1-driven phosphorylation of the other sites (Figure 3F). The RxL2 docking motif enhanced Cdc6 phosphorylation by about 10-fold, indicating that RxL2 directs S-CDK to phosphorylate T7 (Figures S3B and S3C). This suggests that phosphorylation of the N-terminal cluster followed the Cks1-dependent mechanism supported by cyclin docking, similar to that shown previously for Sic1 (Kõivomägi et al., 2011a, Kõivomägi et al., 2013; Figure 3G; Figure S3C). Phosphorylation of the C-terminal degron by S-CDK was much weaker (Figure 3E), and it has been shown to involve the GSK3 kinase Mck1 (Al-Zain et al., 2015). Monitoring the dynamics of Cdc6 versions at G1-S revealed that mutations of the first degron, RxL2, or Cks1 priming sites caused only about a 1-min delay in degradation relative to Whi5 nuclear exit (Figures S3D and S3E). A similar degradation profile was observed with Cdc6(T368A S372A); however, the Cdc6 levels were higher because of more prominent accumulation in mitosis (Figure 3C). These findings suggest that, at G1-S, both degrons act redundantly, whereas regulation of Cdc6 during mitotic exit is carried out by the second degron alone, since the N-terminal degron is shielded by M-CDK.

To demonstrate the shielding of the degron in the inhibitory complex *in vitro*, we performed a kinase assay using M-CDK in a 1:1 ratio with either wild-type Cdc6 or Cdc6(lxf). In the case of wild-type Cdc6, M-CDK phosphorylated mainly one site in Cdc6, and addition of S-CDK did not lead to further phosphorylation (Figure 3H). Contrarily, Cdc6(lxf) was multi-phosphorylated by M-CDK. Importantly, M-CDK itself phosphorylates the degron when the LxF is mutated (Figure S3F). This provides further evidence that the LxF motif is required to shield phosphorylation of the N-terminal degron. Next, to demonstrate the shielding *in vivo*, we used a strain with *CDH1* deletion to follow the G1-S dynamics of Cdc6 in the presence of Clb2 (Figure S3G). Strikingly, *CDH1* deletion strongly stabilized Cdc6(T368A S372A), showing a similar profile as Cdc6(T39A S43A T368A S372A lxf), where both degrons are mutated (Figure 3I). This suggests that, in *cdh1* cells, the N-terminal degron is shielded by Clb2 during the entire cell cycle. However, when the LxF mutation was combined with T368A S372A, Cdc6 degradation was very fast (Figure 3I). In addition to shielding the degron, M-CDK might also sequester Cdc6 from replication proteins, as shown previously (Mimura et al., 2004; Figure 3J). Strains that express Cdc6(lxf) have fewer cells in S phase, which suggests that, in wild-type cells, M-CDK-Cdc6 interaction might inhibit replication origin licensing (Figure S3H).

### The LxF Motif Stimulates Mitotic Exit by Mediating Spo12 Phosphorylation

**Figure 4:**
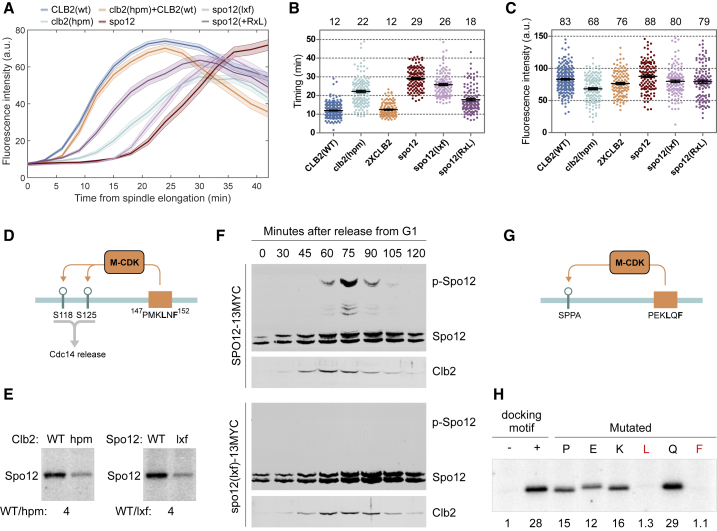
LxF Targets M-CDK for Spo12 Phosphorylation, Leading to FEAR Network Activation (A) The NLS-NES-GFP Cdk1 activity sensor was used to measure the timing of Cdc14 phosphatase activation after spindle elongation in a live cell microscopy assay. This plot shows average nuclear levels of the sensor from anaphase onset in the indicated strains. (B) Measured time periods from spindle elongation to the Cdk1 activity sensor reaching 50% of its G1 nuclear level in single cells. (C) Peak G1 nuclear fluorescence intensities of the sensor in individual cells. The numbers above the plots in (B) and (C) show the average values of the indicated strains. (D) Scheme showing the docking motif and phosphorylation sites in Spo12. (E) Phosphorylation of purified Spo12 by Clb2-Cdk1 is dependent on the LxF. SDS-PAGE phosphoimages are shown. WT/hpm shows the relative phosphorylation rate of Spo12 between Clb2(WT) and Clb2(hpm). WT/lxf shows the effect of LxF mutation. (F) Western blot showing phosphorylation of Spo12 and expression of Clb2 in cells released from α-factor-induced G1 arrest. For Spo12, the lysates were separated using Phos-tag SDS-PAGE, whereas for Clb2, standard SDS-PAGE was used. (G) A model substrate based on Sic1 amino acids 1–33 with a minimal consensus phosphorylation site (SP) at a 24-amino-acid distance from the LxF motif (PEKLQF from Cdc6) was used to study LxF as a linear docking motif. (H) Mapping of the effect of different amino acids near the LxF motif by single alanine mutations. Phosphoimage SDS-PAGE is shown. The numbers below the lanes show the relative phosphorylation rates compared with the substrate without the docking site. See also Figure S4.

We found that nuclear entry of the Cdk1 activity sensor was severely delayed in a *clb2(hpm)* strain (Figures 4A–4C). Such an outstanding effect, compared with the *lxf* mutation in Cdc6 (Figure 2E), raised the possibility that the *hp* of Clb2 mediates other crucial interactions in mitosis. Addition of a wild-type copy of *CLB2* rescued the *clb2(hpm)* phenotype in the sensor localization (Figures 4A–4C). This suggests that the *clb2(hpm)* effect on sensor dynamics is not due to the lack of Cdk1 inhibition but, instead, the absence of LxF-mediated M-CDK activity and, thus, a lower level of mitotic phosphorylation. To find the key targets that cause this effect, we analyzed the disordered regions of mitotic Cdk1 targets and introduced mutations in a number of candidate LxF motifs.

First, we focused on Spo12, whose phosphorylation by M-CDK is essential for the FEAR network (Tomson et al., 2009). Two CDK sites in Spo12 (S118 and S125) have been shown to be necessary for timely release of the phosphatase Cdc14 (Tomson et al., 2009). The distance between an LxF motif and the phosphorylation sites is ideal for docking (Figure 4D) because 20–40 amino acids is the optimal distance between the phosphorylation site and docking motif (Kõivomägi et al., 2013). We found that phosphorylation of purified Spo12 was dependent on *hp* and a conserved LxF motif (Figure 4E; Figure S4A). Strikingly, the mutation of LxF in *SPO12* delayed the sensor to the same extent as with *SPO12* deletion, which suggests that *hp*-dependent phosphorylation of Spo12 by M-CDK in anaphase is essential to activate the FEAR network (Figures 4A and 4B).

To follow the phosphorylation of Spo12 *in vivo*, we used Phos-tag western blotting of synchronized cells. Phosphorylation of Spo12 peaks 75 min after release from G1, at a time when Clb2 levels are already declining, confirming that phosphorylation of Spo12 occurs in anaphase (Figure 4F; Tomson et al., 2009). Interestingly, when we mutated the LxF motif in Spo12, no phosphorylation of Spo12 could be detected (Figure 4F), which indicates that LxF is critical for targeting Spo12 phosphorylation. To confirm that loss of phosphorylation because of the LxF mutation is caused by disrupting the interaction between Clb2 and Spo12, we replaced the LxF motif with a different cyclin docking motif, the RxL motif. This rescued the timing of nuclear import of the Cdk1 activity sensor while still being around 6 min delayed compared with the wild-type strain (Figures 4A and 4B). This could indicate that the RxL motif can direct phosphorylation of Spo12 by RxL-specific Clb3- or Clb5-Cdk1 (the remaining tail of the peak).

These findings confirm the hypothesis that, besides the inhibitory function of LxF in Cdc6, the motif can enhance phosphorylation. To confirm its function as a modular substrate docking motif in the context of a model CDK substrate, we introduced the LxF motif from Cdc6 into Sic1, a well-studied target of Cdk1 (Figure 4G). In a Sic1-based construct that contained only one minimal consensus phosphorylation site, the LxF motif strongly promoted Clb2-dependent phosphorylation (Figure 4H). Mapping in the vicinity of the LxF motif with single alanine mutations confirmed the exclusive importance of L and F amino acids and a minor importance of P in position −3, E in position −2, and K in the −1 position, counted from the leucine in LxF (Figure 4H).

### The LxF Interaction Drives the Regulation of M-CDK by Swe1

In the early cell cycle, Swe1 binds M-CDK to phosphorylate the inhibitory site Y19 in Cdk1, whereas rising CDK activity leads to multi-phosphorylation and inactivation of Swe1 (Harvey et al., 2005). The mechanism that controls the interactions between Swe1 and CDK remains unsolved. It is known that Clb2-Cdk1 has the highest affinity for Swe1 compared with earlier CDK complexes (Hu and Aparicio, 2005, Keaton et al., 2007) and that this depends on the *hp* of Clb2 (Hu et al., 2008).

**Figure 5:**
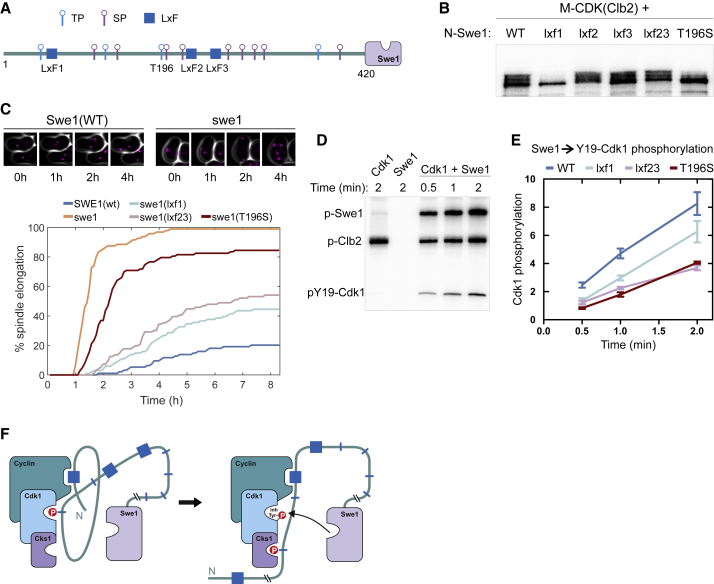
LxF Plays a Key Role in Swe1 and M-CDK Interaction (A) A diagram of candidate LxF motifs and CDK consensus sites in the disordered N-terminal domain of Swe1. (B) Autoradiograph of an *in vitro* phosphorylation assay of the non-catalytic domain of Swe1 using Clb2-Cdk1, separated in SDS-PAGE. (C) The onset of anaphase in strains expressing different Swe1 mutants was followed after release of cells from pheromone-induced G1 arrest to medium containing Lat B. Spindle elongation was determined using Spc42-mCherry. The images are merged from phase-contrast and mCherry channels. The wild-type cell is arrested in metaphase with short spindle; however, the *swe1* cell progresses to a second cell cycle without budding in 4 h in medium containing Lat B. (D) Autoradiograph of the two-way phosphorylation reactions between Swe1 and Clb2-Cdk1. (E) Quantified profiles of Clb2-Cdk1-Y19 phosphorylation in a two-way assay for indicated Swe1 mutants. The error bars show SD. (F) Diagram illustrating the mechanism of the Swe1 and M-CDK interaction. See also Figure S5.

We mutated three candidate LxF motifs in the N-terminal non-kinase domain of Swe1 (N-Swe1 [1–450]) (Figure 5A). A kinase assay using Clb2-Cdk1 revealed that mutation of the most N-terminal motif (LxF1) greatly reduced the phosphorylation shift of N-Swe1, whereas mutations in the other motifs had little effect (Figure 5B). In kinase assays with Clb2(*hpm*), a similar drop in phosphorylation as with the LxF1 mutant was observed (Figure S5A). These results suggest that LxF1 is the major docking motif responsible for Clb2 *hp*-dependent phosphorylation of Swe1.

To test the role of the Swe1 candidate LxF motifs *in vivo*, we set up an assay to measure Swe1-mediated mitotic arrest because of depolymerization of actin in response to treatment with Latrunculin B (Lat B) (McMillan et al., 1998, McMillan et al., 1999). Cells were synchronized by pheromone arrest, and spindle elongation was measured after release from G1 to medium containing Lat B (Figure 5C). Both the N-terminal motif LxF1 and a LxF2 and LxF3 double mutant displayed faster accumulation of anaphase cells compared with the wild-type strain. This suggests that Swe1-dependent inhibition of M-CDK was impaired in both cases. These results, combined with the phosphorylation data in Figure 5B, led us to hypothesize that, as in Cdc6, a threonine-based optimal CDK site just upstream of LxF2 and LxF3, T196, could serve as a Cks1 docking site that, together with the LxF sites, would form a complex presenting CDK for tyrosine phosphorylation by Swe1 (Figure S5B). This idea was supported by the fact that substitution of threonines with serines in key CDK sites in Swe1 reduces tyrosine phosphorylation of Cdk1 *in vivo* (McGrath et al., 2013) and also by the observation that Swe1 mutants lacking catalytic activity exhibit some of the inhibition of Cdk1 (McMillan et al., 1999). The cell cycle progression of the *swe1(T196S)* strain was considerably less hindered upon actin depolymerization compared with the wild-type strain (Figure 5C). These data allow one to argue that the key mechanism for docking M-CDK for tyrosine phosphorylation is binding of phosphorylated T196 with Cks1, supported by the Clb2-LxF interactions.

To test this hypothesis, we performed a two-way assay of Cdk1 and Swe1 mutual phosphorylation using an active Swe1 preparation (Figures 5D and 5E; Figure S5C). The initial velocities of Cdk1 Y19 phosphorylation were lowest in the T196S and LxF2 and LxF3 mutants, whereas Swe1(lxf1) displayed an intermediate rate and wild-type Swe1 the highest rate (Figure 5E). This result, combined with the LxF1 effect on N-Swe1 phosphorylation, suggests that LxF1 could direct phosphorylation of T196, followed by binding of pT196 to Cks1 and secondary docking via LxF2 and LxF3, leading to inhibitory Y19 phosphorylation (Figure 5F). This conclusion is well aligned with a previous model suggesting that Swe1 phosphorylation by Cdk1 takes place in two steps (Harvey et al., 2005). First, initial phosphorylation activates Swe1 for Cdk1 phosphorylation, and second, upon further accumulation of M-CDK, the hyper-phosphorylated forms appear (Asano et al., 2005, Harvey et al., 2005). In conclusion, the mechanism of the Swe1-Cdk1 interactions elucidated here represent a key example of differential roles of LxF motifs in building the CDK thresholds via a self-primed inhibitory mechanism and enhanced phosphorylation.

### The Global Importance of the M-CDK-LxF Interaction

**Figure 6:**
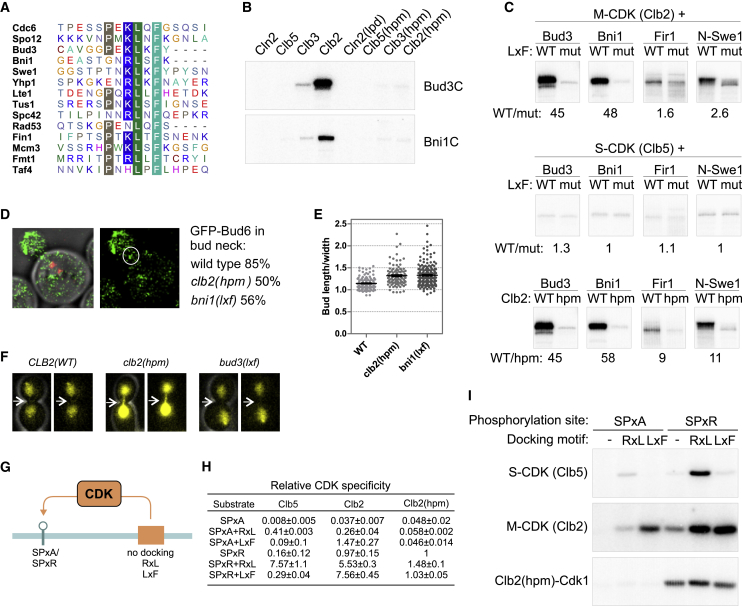
The Global Importance of the LxF Motif in M-CDK Substrate Targeting (A) A sequence alignment of predicted LxF motifs in 14 previously described Cdk1 targets. (B) Autoradiographs showing phosphorylation of the C-terminal domains of Bni1 and Bud3 by different Cdk1 complexes. (C) Autoradiographs of wild-type targets and their LxF mutants from experiments with Clb2-, Clb5-, and Clb2(*hpm*)-Cdk1. For comparison, we analyzed two additional LxF-dependent targets, Fir1 and N-Swe1. WT/mut shows the phosphorylation rate of the wild type and the LxF mutant. WT/hpm denotes the relative phosphorylation rate of wild-type Clb2 and Clb2(*hpm*). (D) Localization of GFP-Bud6 in metaphase cells. SPBs are marked by Spc42-mCherry. The image on the left is merged from bright-field, GFP, and mCherry channels; the image on the right shows the GFP-Bud6 signal. The white oval marks the bud neck. Shown on the right are the percentages of metaphase cells of the indicated strains that have a detectable GFP-Bud6 signal at the bud neck. (E) The bud length to width ratios of metaphase cells are shown for the indicated strains. (F) Localization of Clb2-Citrine in anaphase cells expressing either wild-type Clb2, Clb2(*hpm*), or Bud3 with a mutated LxF motif. The images are merged phase-contrast with the Citrine signal (left) and the Citrine channel alone (right). The white arrow points to the bud neck. (G) Scheme of the Sic1-based model substrates used to analyze the specificities of Cdk1 complexes. (H) Estimated phosphorylation specificities (relative k_cat_/K_M_ values) of the substrates used in (I), calculated as the relative rates divided by enzyme concentration of the reaction performed under initial velocity conditions with non-saturated enzyme. The obtained values were normalized against the substrate with the SPxR site and no docking sites phosphorylated by Clb2(hpm)-Cdk1. (I) Autoradiographs showing *in vitro* phosphorylation of the model substrates with the indicated kinase complexes. See also Figures S6 and S7 and Table S1.

To estimate the global importance of M-CDK docking, we searched for potential LxF motifs from disordered regions of the *S. cerevisiae* proteome. Based on mapping of the LxF motif (Figure 4H) and the conservation of LxF motifs in Cdc6, Swe1, and Spo12 (Figures S4A–S4C), we searched for motifs where, in addition to the essential L and F, at least two of three residues in positions −3 to −1 from L also matched the consensus (P in −3, N or E in −2, K or R in −1). This motif is present in 72 proteins (Table S1), 14 of which have been identified as Cdk1 targets in large screens (Figure 6A; Ubersax et al., 2003, Holt et al., 2009).

We purified two of these targets, Bud3 and Bni1, and analyzed the cyclin specificity of their phosphorylation. Both Bud3 and Bni1 are highly Clb2-specific substrates, and notably, mutation of the *hp* of Clb2 or the predicted LxF motif in substrates decreases their phosphorylation rate by over 40-fold (Figures 6B and 6C). Importantly, the LxF motif promotes phosphorylation specifically in the case of M-CDK but not for S-CDK (Figure 6C). Formin Bni1 interacts with a nucleation-promoting factor and the polarity determinant Bud6, which is localized at the bud tip and bud neck (Segal et al., 2000). Both Bni1 and Bud6 are involved in polarized growth and mitotic spindle orientation (Segal et al., 2000, Graziano et al., 2011). The relative bud tip or neck distribution of Bud6 is dependent on Bni1, and accumulation of Bud6 starts in mitosis (Segal et al., 2000). The C-terminal domain of Bni1 is necessary for interaction with Bud6 (Evangelista et al., 1997) and contains 10 CDK phosphorylation sites and an LxF motif. This led us to hypothesize that phosphorylation of Bni1 by M-CDK might localize Bud6 to the bud neck. To test this, we analyzed the localization of GFP-Bud6. GFP-Bud6 was detectable at both the bud neck and bud tip in 85% of wild-type metaphase cells; however, in *clb2(hpm)* or *bni1(lxf)* cells, GFP-Bud6, although still detectable at the bud tip, was localized to the bud neck in less than 60% of metaphase cells (Figure 6D; Figure S6A). Also, phosphorylation of Bni1 could be involved in growth depolarization because both *clb2(hpm)*- and *bni1(lxf)*- expressing cells have slightly elongated buds in the *GFP-BUD6* strain (Figure 6E).

Bud3 has been shown to recruit Clb2 to the bud neck in a *hp*-dependent manner for timely phosphorylation and degradation of Swe1 (Bailly et al., 2003, Kao et al., 2014). To test whether the LxF motif in Bud3 mediated this interaction, we analyzed the localization of Clb2-Citrine. In wild-type cells, a faint Clb2-Citrine signal could be seen at the bud neck in anaphase cells; however, no bud neck localization of Clb2 could be detected in cells carrying either *bud3(lxf)* or *clb2(hpm)* (Figure 6F; Figures S6B–S6D).

### A Quantitative Model of Cyclin Specificity

Our study adds the final missing element, the M-CDK-specific docking mechanism, to the cyclin specificity model. Previously, docking mechanisms for three of the four major classes of budding yeast CDK complexes have been found: G1-CDK (Bhaduri and Pryciak, 2011, Kõivomägi et al., 2011b), S-CDK (Wilmes et al., 2004, Loog and Morgan, 2005), and G2-CDK (M.Ö. and M.L., unpublished data). We have also shown that cyclins modulate the intrinsic activity (k_cat_/K_M_) of Cdk1 in an increasing order corresponding to their appearance in the cell cycle: G1-CDK < S-CDK < G2-CDK < M-CDK (Kõivomägi et al., 2011b). For a full quantitative model of specificity, we developed a set of substrates with either a full or minimal consensus phosphorylation site located at its N-terminal part and cyclin docking motifs at the C terminus (Figure 6G).

The most prominent effect of adding the LxF motif was a 40-fold increase in k_cat_/K_M_ values in the case of a minimal consensus site (Figures 6H and 6I). For the full consensus site, the effect was close to 7-fold, which agrees with our previous observations that M-CDK has a low micromolar K_M_ value for full consensus sites, leaving less space for potentiation from the docking interactions, whose affinity is in the same range (Kõivomägi et al., 2011b). As expected, the RxL motif strongly enhanced the optimal site phosphorylation by S-CDK, whereas the LxF motif showed only a very mild effect in this context, confirming the different specificity (Figures 6H and 6I).

## Discussion

In the present study, we characterized a cyclin-specific docking motif, the LxF motif, that binds the mitotic cyclins Clb1 and Clb2 but does not bind the G1, S, and G2 cyclins (Cln2, Clb5, and Clb3, respectively). This motif was first mapped in the replication licensing factor Cdc6 while exploring its contribution to an inhibitory mechanism initiated via priming of Cdc6 for the phospho-adaptor Cks1. This mechanism leads to shielding of the Cdc6 N-terminal degron by M-CDK, which would also prevent Cdc6 from loading replication origins before G1, as shown previously by Mimura et al. (2004). Furthermore, these mechanisms might be critical in meiosis. Clb1 is not degraded during transition from meiosis I to meiosis II (Carlile and Amon, 2008, Phizicky et al., 2018), and, unlike in mitosis, there is no origin licensing between the two divisions. Although this is guaranteed by multiple factors, including the inability to fully suppress Cdk1 activity, there is still notable Cdc6 accumulation between anaphase I and meiosis II (Phizicky et al., 2018). Therefore, after the first meiotic division, tight interaction between Cdc6 and Clb1-Cdk1 could function as an additional control mechanism that sequesters Cdc6 to prevent origin licensing between the two meiotic divisions.

Interestingly, based on homology and conservation of the *hp* of cyclins, Archambault et al. (2005) proposed that yeast mitotic cyclins have evolved a different binding pocket compared with S-cyclins because of the *hp* being different but still conserved among M-cyclins. The work presented here has shown that, indeed, M-CDK has minor specificity toward the S-CDK specific RxL motif; however, it has evolved to bind a different M-CDK specific motif (Figures S7A and S7B). In both mammalian and yeast cells, the RxL motif strongly potentiates phosphorylation by S-CDKs but has a lesser effect on M-CDKs (Figures 6H and 6I; Cheng et al., 2006, Brown et al., 2007, Petri et al., 2007). Interestingly, although the *hp* of mammalian cyclin B and yeast Clb2 are not identical, there are differences in the same positions in M-cyclins compared with S-cyclins (Figures S7C and S7D), indicating that there could also be M-CDK-specific docking motifs in higher eukaryotes.

In addition, the LxF motif was found to have a wider role in phosphorylation of mitotic targets. A key target, Spo12, which controls release of the mitotic exit phosphatase Cdc14, was found to be phosphorylated late in mitosis in an LxF-dependent manner. We also discovered that different LxF motifs and Cks1-dependent priming interactions were behind the mechanism of M-CDK inhibitory phosphorylation by Swe1. In addition, many potential LxF motifs were found in CDK targets, and a number of these were demonstrated to be functional.

### A Unified Model of CDK Function: Thresholds and Cyclin Specificity

**Figure 7:**
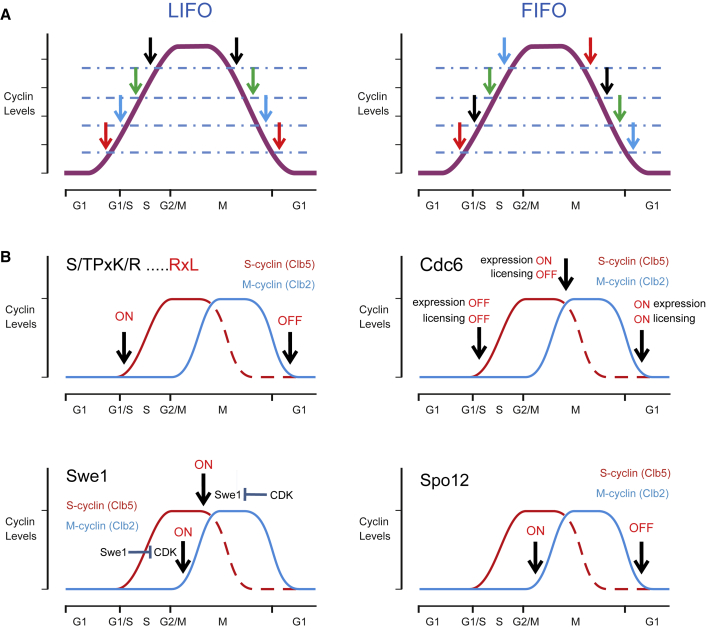
Different Switching Orders Are Generated by Combining the LxF, RxL, and Cks1 Docking Mechanisms (A) In a simple model with uniform CDK activity that triggers the cell cycle switches at different activity thresholds, the switching order would follow the principle of last in, first out (LIFO). Alternatively, the first in, first out order (FIFO, right panel) would provide more complexity in temporal switching. (B) Cyclin-specific targeting and Cks1-mediated docking create a variety of possible thresholds and switching orders (combinations of LIFO and FIFO), as exemplified using the CDK targets studied in the current paper. Top left: the substrate type with a full CDK consensus site and S-CDK-specific RxL motif would provide a low switching threshold that would be useful for targets whose phosphorylation status should be kept constant from the beginning of the cell cycle (ON) until the end of mitosis (OFF). Top right: the Cdc6 mechanism provides a set of three switches involving (1) S-CDK-specific degron phosphorylation at the onset of S phase (Cdc6 expression OFF, licensing OFF); (2) at the beginning of mitotic exit, M-CDK docking of Cdc6 via the LxF motif and Cks1 (Cdc6 expression ON, licensing OFF via shielding); and (3) at the onset of G1 (Cdc6 expression ON, licensing ON). Bottom left: the double-negative feedback mechanism between M-CDK and Swe1 is using multiple LxF motifs and the Cks1 mechanism to introduce two thresholds: (1) M-CDK phosphorylation to prime the site T196, leading to Cks1- and LxF-mediated inhibitory phosphorylation of CDK; (2) multi-phosphorylation of Swe1 and relieving the CDK inhibition. Bottom right: M-CDK-specific phosphorylation of Spo12 via the LxF-*hp* mechanism provides one high (ON) and one low (OFF) threshold, considering the net CDK activity profile.

For a systems-level understanding of the role of cyclin specificity, one can apply the concept of single input module (SIM), which is used in transcriptional networks to order the sequential activation of genes given the gradual accumulation of a common input signal (Alon, 2007). A simple SIM with gradually increasing levels of an activator that meets the increasing activation thresholds is analogous to the uniform accumulation of CDK activity during the cell cycle of a possible single cyclin-CDK system and can create a so-called last in, first out (LIFO) switching order (Figure 7A). In this case, however, the mitotic events triggered at the highest CDK thresholds receive a reverse upstream signal immediately in anaphase when CDK activity begins to drop. Conversely, a system of different cyclins with unique specificity and Cks1-dependent enhancing or diverting mechanisms provides more complex switching orders, including combinations of LIFO and its alternative, first in, first out (FIFO; the circular queue), orders (Figures 7A and 7B). The sequential waves of cyclin-CDK complexes with changing docking specificity and increasing intrinsic activity would provide a wider combination of switching orders and thresholds compared with the uniform specificity. Indeed, mitotic events triggered at higher thresholds may be required to stay in the ON state until the end of mitotic exit. Thus, cyclin-specific substrate recognition can provide two or more different thresholds for one target. In addition, by combining cyclin specificity with Cks1-mediated mechanisms, even more complex sets of thresholds can be assigned to a target, as exemplified based on the targets studied in this paper (Figure 7B). Another level of complexity in CDK thresholds comes from the differential activity of counteracting phosphatases. For example, the mitotic exit phosphatase Cdc14 preferentially dephosphorylates SP sites, and it was recently discovered that a linear PxL motif on CDK targets directs the Cdc14 activity to specific substrates (Bremmer et al., 2012, Kataria et al., 2018). In conclusion, the CDK threshold model and the cyclin specificity model are not alternatives for each other; instead, our data lead to a unified model of CDK function according to which cyclin specificity and Cks1 mechanisms provide ways to flexibly create more thresholds for CDK and optimize the cell cycle process. The unified model does not conflict with the single mitotic cyclin system because M-CDK, although it has the strongest intrinsic activity on the active-site level (Loog and Morgan, 2005, Kõivomägi et al., 2011b), can maintain robust ordering of the switches (Örd and Loog, 2019). The cyclin-specific and Cks1 mechanisms, however, provide fine-tuning of the threshold ladder, which would assume critical importance when competitive fitness becomes crucial for survival.

## STAR★Methods

### Key Resources Table

**Table:** 

REAGENT or RESOURCE	SOURCE	IDENTIFIER
**Antibodies**		

anti-Myc (9E10)	Santa Cruz Biotechnology	sc-40; RRID: AB_627268
anti-Clb2	Santa Cruz Biotechnology	sc-9071; RRID: AB_667962
anti-HA	Labas AS	N/A
Goat anti-rabbit, HRP-conjugated	Labas AS	N/A
Goat anti-mouse, HRP-conjugated	Labas AS	N/A

**Chemicals, Peptides, and Recombinant Proteins**		

Latrunculin B	Tocris	3974
α-factor peptide (WHWLQLKPGQPMY)	ProImmune	N/A
HA peptide (CYPYDVPDYAGYPYDVPDYAG)	ProImmune	N/A
Phos-tag Acrylamide AAL-107	Wako Chemicals	304-93521
ATP, [γ-32P]-10mCi/ml	Hartmann Analytical	SRP-501
SuperSignal West Pico PLUS Chemiluminescent Substrate	ThermoFisher Scientific	34577
Bio-Rad Protein Assay Dye Reagent Concentrate	Bio-rad	#5000006

**Deposited Data**		

Unprocessed autoradiographs and western blot images of this study	This study	https://doi.org/10.17632/r7hv4zj6zw.1

**Experimental Models: Organisms/Strains**		

*S. cerevisiae* strains used in this study were in W303 or BY4741 background and are listed in Table S2.		N/A

**Recombinant DNA**		

Plasmids used in the study are listed in Table S3		N/A

**Software and Algorithms**		

MATLAB scripts for cell tracking and quantification of fluorescence signals	Doncic et al., 2013	N/A
SLiMSearch4	Krystkowiak and Davey, 2017	http://slim.ucd.ie/slimsearch/
PSIPRED v3.3	(Buchan et al., 2013)	http://bioinf.cs.ucl.ac.uk/psipred/
UCSF Chimera	(Pettersen et al., 2004)	https://www.cgl.ucsf.edu/chimera/

### Contact for Reagent and Resource Sharing

Further information and requests for resources and reagents should be directed to and will be fulfilled by the Lead Contact, Mart Loog (mart.loog@ut.ee).

### Experimental Model and Subject Details

#### Yeast strains and media

Yeast strains were haploid derivates of the W303 strain and are described in Table S2. Gene deletions, promoter substitutions and epitope-tagging were performed using PCR-based homologous recombination (Longtine et al., 1998, Janke et al., 2004). The NLS-NES module based on Mcm2 and Mcm3 (Liku et al., 2005) was expressed under an *ADH1* promoter and was C-terminally tagged with EGFP. The ADH1-NLS-NES-GFP cassette was integrated into the genome at the genomic *ADH1* promoter locus using an integration plasmid. In plots where Cdc6 mutants are used, expression of genomic *CDC6* was controlled by a *GALS* promoter and an additional copy of the *CDC6* gene with *CDC6* promoter was integrated to the genome at the genomic *URA3* locus using an pRS306-based plasmid. The transformants were selected for single-copy integration by fluorescence intensity. The recombinant proteins were expressed in *E. coli* strain BL21-CodonPlus(DE3)-RP cells that were grown in 2xYT media.

### Method Details

#### Sic1-based substrate constructs

In studies that analyzed the LxF motif in substrate docking we used substrate constructs based on the N terminus of Sic1. The Sic1 sequence from positions 1-33 with mutations T2A and T5S followed by a linker with sequence ELQGGGGG was fused with immunoglobulin-binding domain of streptococcal protein G (GB1 domain) containing a C-terminal 6xHis tag. In constructs with cyclin docking motifs, amino acids in positions 27-33 were replaced with either the sequence PEKLQF from Cdc6 (LxF motif) or with the sequence VNRILFP from Sic1 (RxL motif).

#### Protein purification

Clb5-, Clb3-, Clb2-Cdk1 complexes and full-length Swe1 were purified from yeast cells using TAP method with C-terminally tagged cyclins as described previously (Puig et al., 2001, Ubersax et al., 2003), except that lysates were prepared using Mixer Mill MM 400 (Retch). For purification of Cln2-Cdk1 complexes, we used Cln2 N-terminally tagged with 3HA that was overexpressed in yeast cells and purified by immunoaffinity chromatography with an antibody against the HA epitope as described previously (McCusker et al., 2007) (rabbit polyclonal anti-HA was purchased from Labas AS, Estonia). Cks1 was purified as described in (Reynard et al., 2000).

N-terminal 6xHis-tag was used for purification of Cdc6, Swe1(1-450) and Fir1. 6His-tagged substrates were expressed in *E. coli* BL21RP at 23°C using 0.3 mM IPTG. Sic1-GB1-6xHis-tag substrate constructs were expressed in *E. coli* BL21RP at 37°C using 1 mM IPTG. His-tagged proteins were purified by standard cobalt affinity chromatography with 200 mM imidazole used for elution. GST-Spo12, GST-Bud3(1222-1636) and GST-Bni1(1215-1953) were expressed in *E. coli* BL21Rosetta at 18°C using 0.3 mM IPTG and were purified using glutathione-Sepharose beads (GE Healthcare). Cells were lysed using lysozyme, except in case of Swe1(1-450), the lysate was prepared using Mixer Mill MM 400 (Retch).

#### Kinase assays

The general composition of the assay mixture contained 50 mM HEPES, pH 7.4, 150 mM NaCl, 5 mM MgCl_2_, 20 mM imidazole, 2% glycerol, 0.2 mg/ml BSA, 500 nM Cks1 and 500 μM ATP [(with added [γ-32P]-ATP (Hartmann Analytic)].

To analyze the inhibition of Cdk1 activity by Cdc6, we used bovine histone H1 (Sigma-Aldrich) as a reference substrate (final concentration 80 μg/ml). In the inhibition assays, we used 7-10 reactions with Cdc6 concentrations ranging from 0.1 nM to 3 μM and two reactions containing no Cdc6. The concentration of the Clb2-Cdk1 complex was 0.2 nM and reactions were started by mixing the enzyme with a solution containing Cdc6 and histone H1. Reactions were carried out at room temperature and were stopped using SDS-PAGE sample buffer at the 12-minute time point.

For the phosphorylation assays, substrate protein concentrations were in the range of 1-2 μM (in the linear [S] versus v_0_ range, several-fold below the estimated K_M_ value). The concentrations of kinase complexes were 0.2-2 nM. The kinase assays were performed under conditions below 10% of the initial substrate turnover. Reactions were stopped using SDS-PAGE sample buffer.

In the assay analyzing the shielding of degron by Clb2-Cdk1 from Clb5-Cdk1 *in vitro* (Figure 3H), Cdc6 and Clb2-Cdk1 were both at 10 nM concentration. Also, to enhance the detection of 32P-phosphorylation signals, ATP concentration in these assays was lowered to 250 μM. Clb5-Cdk1 was used in 4 nM concentration. The reaction was stopped at the 3-minute time point using SDS sample buffer. To separate the phosphorylated forms of Cdc6, we used 7.5% SDS-PAGE supplemented with 25 μM Phos-tag reagent (Wako Pure Chemical Industries). Electrophoresis was performed at 15 mA for 2.5 h.

The two-way phosphorylation assay between Cdk1 and Swe1 was performed using 6.5 nM Clb2-Cdk1 and 0.65 nM Swe1. The reaction was stopped at 30 s, 1 min, and 2 min. To be able to detect phosphorylation signals under initial velocity conditions, ATP concentrations were lowered to 10 μM to incorporate more 32P-ATP.

Radioactivity was detected using Typhoon Trio (Amersham Biosciences) and quantification of signals was performed using ImageQuant TL (Amersham Biosciences). GraphPad Prism 5.0 was used for data analysis.

#### Time-lapse fluorescence microscopy

Yeast cultures were grown at 30°C in synthetic complete media with 2% glucose (SC) to OD 0.2-0.6. Cells were pipetted onto 0.8 mm coverglass and covered with a 2% agarose pad made with SC. Before imaging, the cells were allowed to proliferate on the slide for 60 min. Imaging was performed at 30°C using a Zeiss Observer Z1 microscope with a 63X/1.4NA oil immersion objective and Orca-r2 C10600-10B camera (Hamatsu Photonics). Temperature of the agarose pad was held at 30°C using Tempcontrol 37-2 digital from PeCon. Images were taken every 3 min and imaging sessions were 8 h long. Up to 12 positions were imaged using an automated stage and Axiovision software. Definite Focus was used to keep the cells in focus during time-lapse microscopy. NLS-NES-GFP expressing strains were exposed for 15 ms using a Colibri 470 LED module. Clb2- and Cdc6-yeCitrine strains were exposed for 250 ms using Colibri 505 LED module. Spc42-mCherry was imaged using Colibri 540-580 LED module for 750 ms. All Colibri modules were used at 25% power. Image segmentation, cell tracking and quantification of nuclear fluorescence signals was performed using MATLAB (The MathWorks, Inc.) as described in Doncic et al. (2013). For every strain, data are from at least two repeats with different transformants.

#### Western blotting

Spo12-13MYC expressing cells were grown in YPD at 30°C to OD 0.3, then treated for 2.5 hr with 1 μg/ml α-factor and released into fresh medium. Cells were lysed by bead beating in a lysis buffer containing urea. For analysis of Spo12 phosphorylation, proteins were separated using Phos-tag SDS-PAGE with 50 μM Phos-tag and 8% acrylamide. Blotting of Phos-tag SDS-PAGE gels was performed using a dry system iBlot (Invitrogen). Blotting of SDS-PAGE gels was carried out using a Pierce G2 Fast Blotter (Thermo Scientific). c-Myc monoclonal antibody (1:500) (9E10, Santa Cruz Biotechnology) and HRP-conjugated anti-mouse antibody (1:7500) from Labas, Estonia were used to detect MYC-tagged proteins by western blotting. To detect Clb2 protein, we used rabbit polyclonal Clb2 antibody (1:500) (y-180, Santa Cruz Biotechnology) and HRP-conjugated anti-rabbit antibody (1:7500) from Labas, Estonia.

#### FACS analysis

Yeast cultures were grown overnight at 30°C in synthetic complete media with 2% glucose (SC) to OD 0.6. Cultures were pipetted into 80% ethanol and cells were fixed overnight in a rotator at 4°C. Fixed cells were treated with RNase A (Sigma) (1 mg/ml RNase A for 40 min at 37°C) and Proteinase K (Thermo Scientific) (200 μg/ml Proteinase K for 1h at 37°C). DNA was stained with SYBR Green I (Sigma-Aldrich) for 1 h.

#### Bioinformatics analysis

SLiMSearch4 (Krystkowiak and Davey, 2017) was used to search the disordered regions of *S. cerevisiae* proteome for potential LxF docking motifs. The IUPRED disorder cut-off score was 0.3. Based on mutational mapping of the motif in Cdc6 and conservation of the motifs, we searched for 3 motifs: P.[KR]L.F, [NE][KR]L.F and P[NE].L.F (where ‘.’ is any amino acid). The hits from these 3 searches were gathered as potential LxF motifs.

Protein secondary structure predictions were performed using PSIPRED v3.3 (http://bioinf.cs.ucl.ac.uk/psipred/).

The phylogenetical analysis of mitotic cyclins in Figure S7B was done using the Phylogeny.fr platform One-Click pipeline (Dereeper et al., 2008).

Structural analysis and alignment of cyclin sequences to human cyclin A2 structure (2CCI; Cheng et al., 2006) was carried out in UCSF Chimera (Pettersen et al., 2004).

### Quantification and Statistical Analysis

The data from time-lapse microscopy experiments is from at least two replicate experiments with different transformants in case of Cdc6 mutant strains. All replicate experiments are included in the data. The statistical details of the experiments can be found in the figure legends, the exact number of cells used in microscopy data is presented in Table S4.

### Data and Software Availability

Unprocessed autoradiographs and western blot images of this study are available at this link: https://doi.org/10.17632/r7hv4zj6zw.1.

## Supplemental Information

Document S1. Figures S1–S7 and Tables S1–S4

Document S2. Article plus Supplemental Information

## References

[bib1] Al-Zain, A., Schroeder, L., Sheglov, A., and Ikui, A.E. (2015). Cdc6 degradation requires phosphodegron created by GSK-3 and Cdk1 for SCFCdc4 recognition in Saccharomyces cerevisiae. Mol. Biol. Cell 26, 2609-2619. 10.1091/mbc.E14-07-1213 PMC4501359 25995377

[bib2] Alon, U. (2007). An Introduction to Systems Biology: Design Principles of Biological Circuits (Chapman & Hall/CRC).

[bib3] Archambault, V., Li, C.X., Tackett, A.J., Wasch, R., Chait, B.T., Rout, M.P., and Cross, F.R. (2003). Genetic and biochemical evaluation of the importance of Cdc6 in regulating mitotic exit. Mol. Biol. Cell 14, 4592-4604. 10.1091/mbc.E03-06-0384 PMC313736 12960422

[bib4] Archambault, V., Buchler, N.E., Wilmes, G.M., Jacobson, M.D., and Cross, F.R. (2005). Two-faced cyclins with eyes on the targets. Cell Cycle 4, 125-130. 10.4161/cc.4.1.1402 15611618

[bib5] Asano, S., Park, J.-E., Sakchaisri, K., Yu, L.-R., Song, S., Supavilai, P., Veenstra, T.D., and Lee, K.S. (2005). Concerted mechanism of Swe1/Wee1 regulation by multiple kinases in budding yeast. EMBO J. 24, 2194-2204. 10.1038/sj.emboj.7600683 PMC1150880 15920482

[bib6] Bailly, E., Cabantous, S., Sondaz, D., Bernadac, A., and Simon, M.-N. (2003). Differential cellular localization among mitotic cyclins from Saccharomyces cerevisiae: a new role for the axial budding protein Bud3 in targeting Clb2 to the mother-bud neck. J. Cell Sci. 116, 4119-4130. 10.1242/jcs.00706 12972503

[bib7] Bhaduri, S., and Pryciak, P.M. (2011). Cyclin-specific docking motifs promote phosphorylation of yeast signaling proteins by G1/S Cdk complexes. Curr. Biol. 21, 1615-1623. 10.1016/j.cub.2011.08.033 PMC3196376 21945277

[bib8] Bremmer, S.C., Hall, H., Martinez, J.S., Eissler, C.L., Hinrichsen, T.H., Rossie, S., Parker, L.L., Hall, M.C., and Charbonneau, H. (2012). Cdc14 phosphatases preferentially dephosphorylate a subset of cyclin-dependent kinase (Cdk) sites containing phosphoserine. J. Biol. Chem. 287, 1662-1669. 10.1074/jbc.M111.281105 PMC3265848 22117071

[bib9] Brown, N.R., Lowe, E.D., Petri, E., Skamnaki, V., Antrobus, R., Johnson, L., and Johnson, L.N. (2007). Cyclin B and cyclin A confer different substrate recognition properties on CDK2. Cell Cycle 6, 1350-1359. 10.4161/cc.6.11.4278 17495531

[bib10] Buchan, D.W.A., Minneci, F., Nugent, T.C.O., Bryson, K., and Jones, D.T. (2013). Scalable web services for the PSIPRED Protein Analysis Workbench. Nucleic Acids Res. 41, W349-57. 10.1093/nar/gkt381 PMC3692098 23748958

[bib11] Calzada, A., Sanchez, M., Sanchez, E., and Bueno, A. (2000). The stability of the Cdc6 protein is regulated by cyclin-dependent kinase/cyclin B complexes in Saccharomyces cerevisiae. J. Biol. Chem. 275, 9734-9741. 10.1074/jbc.275.13.9734 10734126

[bib12] Calzada, A., Sacristan, M., Sanchez, E., and Bueno, A. (2001). Cdc6 cooperates with Sic1 and Hct1 to inactivate mitotic cyclin-dependent kinases. Nature 412, 355-358. 10.1038/35085610 11460169

[bib13] Carlile, T.M., and Amon, A. (2008). Meiosis I is established through division-specific translational control of a cyclin. Cell 133, 280-291. 10.1016/j.cell.2008.02.032 PMC2396536 18423199

[bib14] Cheng, K.-Y., Noble, M.E.M., Skamnaki, V., Brown, N.R., Lowe, E.D., Kontogiannis, L., Shen, K., Cole, P.A., Siligardi, G., and Johnson, L.N. (2006). The role of the phospho-CDK2/cyclin A recruitment site in substrate recognition. J. Biol. Chem. 281, 23167-23179. 10.1074/jbc.M600480200 16707497

[bib15] Coudreuse, D., and Nurse, P. (2010). Driving the cell cycle with a minimal CDK control network. Nature 468, 1074-1079. 10.1038/nature09543 21179163

[bib16] Dahmann, C., and Futcher, B. (1995). Specialization of B-type cyclins for mitosis or meiosis in S. cerevisiae. Genetics 140, 957-963. 10.1093/genetics/140.3.957 PMC1206679 7672594

[bib17] De Wulf, P., Montani, F., and Visintin, R. (2009). Protein phosphatases take the mitotic stage. Curr. Opin. Cell Biol. 21, 806-815. 10.1016/j.ceb.2009.08.003 19767188

[bib18] Dereeper, A., Guignon, V., Blanc, G., Audic, S., Buffet, S., Chevenet, F., Dufayard, J.F., Guindon, S., Lefort, V., Lescot, M., et al. (2008). Phylogeny.fr: robust phylogenetic analysis for the non-specialist. Nucleic Acids Res. 36, W465-9. 10.1093/nar/gkn180 PMC2447785 18424797

[bib19] Doncic, A., Eser, U., Atay, O., and Skotheim, J.M. (2013). An algorithm to automate yeast segmentation and tracking. PLoS One 8, e57970. 10.1371/journal.pone.0057970 PMC3592893 23520484

[bib20] Drury, L.S., Perkins, G., and Diffley, J.F.X. (2000). The cyclin-dependent kinase Cdc28p regulates distinct modes of Cdc6p proteolysis during the budding yeast cell cycle. Curr. Biol. 10, 231-240. 10.1016/s0960-9822(00)00355-9 10712901

[bib21] Eluere, R., Offner, N., Varlet, I., Motteux, O., Signon, L., Picard, A., Bailly, E., and Simon, M.-N. (2007). Compartmentalization of the functions and regulation of the mitotic cyclin Clb2 in S. cerevisiae. J. Cell Sci. 120, 702-711. 10.1242/jcs.03380 17264146

[bib22] Enserink, J.M., and Kolodner, R.D. (2010). An overview of Cdk1-controlled targets and processes. Cell Div. 5, 11. 10.1186/1747-1028-5-11 PMC2876151 20465793

[bib23] Evangelista, M., Blundell, K., Longtine, M. S., Chow, C. J., Adames, N., Pringle, J. R., Peter, M., and Boone, C. (1997). Bni1p, a yeast Formin linking Cdc42p and the actin cytoskeleton during polarized morphogenesis. Science 276, 118-122. 10.1126/science.276.5309.118 9082982

[bib24] Godfrey, M., Touati, S.A., Kataria, M., Jones, A., Snijders, A.P., and Uhlmann, F. (2017). PP2ACdc55 phosphatase imposes ordered cell-cycle phosphorylation by opposing threonine phosphorylation. Mol. Cell 65, 393-402.e3. 10.1016/j.molcel.2016.12.018 PMC5296252 28132839

[bib25] Graziano, B.R., DuPage, A.G., Michelot, A., Breitsprecher, D., Moseley, J.B., Sagot, I., Blanchoin, L., and Goode, B.L. (2011). Mechanism and cellular function of Bud6 as an actin nucleation-promoting factor. Mol. Biol. Cell 22, 4016-4028. 10.1091/mbc.E11-05-0404 PMC3204064 21880892

[bib26] Harvey, S.L., Charlet, A., Haas, W., Gygi, S.P., and Kellogg, D.R. (2005). Cdk1-dependent regulation of the mitotic inhibitor Wee1. Cell 122, 407-420. 10.1016/j.cell.2005.05.029 16096060

[bib27] Holt, L.J., Tuch, B.B., Villen, J., Johnson, A.D., Gygi, S.P., and Morgan, D.O. (2009). Global analysis of Cdk1 substrate phosphorylation sites provides insights into evolution. Science 325, 1682-1686. 10.1126/science.1172867 PMC2813701 19779198

[bib28] Hu, F., and Aparicio, O.M. (2005). Swe1 regulation and transcriptional control restrict the activity of mitotic cyclins toward replication proteins in Saccharomyces cerevisiae. Proc. Natl. Acad. Sci. USA 102, 8910-8915. 10.1073/pnas.0406987102 PMC1157011 15956196

[bib29] Hu, F., Gan, Y., and Aparicio, O.M. (2008). Identification of Clb2 residues required for Swe1 regulation of Clb2-Cdc28 in Saccharomyces cerevisiae. Genetics 179, 863-874. 10.1534/genetics.108.086611 PMC2429880 18558651

[bib30] Janke, C., Magiera, M.M., Rathfelder, N., Taxis, C., Reber, S., Maekawa, H., Moreno-Borchart, A., Doenges, G., Schwob, E., Schiebel, E., and Knop, M. (2004). A versatile toolbox for PCR-based tagging of yeast genes: new fluorescent proteins, more markers and promoter substitution cassettes. Yeast 21, 947-962. 10.1002/yea.1142 15334558

[bib31] Kao, L., Wang, Y.-T., Chen, Y.-C., Tseng, S.-F., Jhang, J.-C., Chen, Y.-J., and Teng, S.-C. (2014). Global analysis of cdc14 dephosphorylation sites reveals essential regulatory role in mitosis and cytokinesis. Mol. Cell. Proteomics 13, 594-605. 10.1074/mcp.M113.032680 PMC3916656 24319056

[bib32] Kataria, M., Mouilleron, S., Seo, M.-H., Corbi-Verge, C., Kim, P.M., and Uhlmann, F. (2018). A PxL motif promotes timely cell cycle substrate dephosphorylation by the Cdc14 phosphatase. Nat. Struct. Mol. Biol. 25, 1093-1102. 10.1038/s41594-018-0152-3 PMC6292506 30455435

[bib33] Keaton, M.A., Bardes, E.S.G., Marquitz, A.R., Freel, C.D., Zyla, T.R., Rudolph, J., and Lew, D.J. (2007). Differential susceptibility of yeast S and M phase CDK complexes to inhibitory tyrosine phosphorylation. Curr. Biol. 17, 1181-1189. 10.1016/j.cub.2007.05.075 PMC2034293 17614281

[bib34] Koivomagi, M., and Skotheim, J.M. (2014). Docking interactions: cell-cycle regulation and beyond. Curr. Biol. 24, R647-R649. 10.1016/j.cub.2014.05.060 PMC6771416 25050961

[bib35] Koivomagi, M., Valk, E., Venta, R., Iofik, A., Lepiku, M., Balog, E.R.M., Rubin, S.M., Morgan, D.O., and Loog, M. (2011a). Cascades of multisite phosphorylation control Sic1 destruction at the onset of S phase. Nature 480, 128-131. 10.1038/nature10560 PMC3228899 21993622

[bib36] Koivomagi, M., Valk, E., Venta, R., Iofik, A., Lepiku, M., Morgan, D.O., and Loog, M. (2011b). Dynamics of Cdk1 substrate specificity during the cell cycle. Mol. Cell 42, 610-623. 10.1016/j.molcel.2011.05.016 PMC3115021 21658602

[bib37] Koivomagi, M., Ord, M., Iofik, A., Valk, E., Venta, R., Faustova, I., Kivi, R., Balog, E.R.M., Rubin, S.M., and Loog, M. (2013). Multisite phosphorylation networks as signal processors for Cdk1. Nat. Struct. Mol. Biol. 20, 1415-1424. 10.1038/nsmb.2706 PMC3855452 24186061

[bib38] Krystkowiak, I., and Davey, N.E. (2017). SLiMSearch: a framework for proteome-wide discovery and annotation of functional modules in intrinsically disordered regions. Nucleic Acids Res. 45 (W1), W464-W469. 10.1093/nar/gkx238 PMC5570202 28387819

[bib39] Liku, M.E., Nguyen, V.Q., Rosales, A.W., Irie, K., and Li, J.J. (2005). CDK phosphorylation of a novel NLS-NES module distributed between two subunits of the Mcm2-7 complex prevents chromosomal rereplication. Mol. Biol. Cell 16, 5026-5039. 10.1091/mbc.E05-05-0412 PMC1237101 16093348

[bib40] Longtine, M.S., McKenzie, A., 3rd, Demarini, D.J., Shah, N.G., Wach, A., Brachat, A., Philippsen, P., and Pringle, J.R. (1998). Additional modules for versatile and economical PCR-based gene deletion and modification in Saccharomyces cerevisiae. Yeast 14, 953-961. 10.1002/(SICI)1097-0061(199807)14:10<953::AID-YEA293>3.0.CO;2-U 9717241

[bib41] Loog, M., and Morgan, D.O. (2005). Cyclin specificity in the phosphorylation of cyclin-dependent kinase substrates. Nature 434, 104-108. 10.1038/nature03329 15744308

[bib42] Machu, C., Eluere, R., Signon, L., Simon, M.-N., de La Roche Saint-Andre, C., and Bailly, E. (2014). Spatially distinct functions of Clb2 in the DNA damage response. Cell Cycle 13, 383-398. 10.4161/cc.27354 PMC3956534 24300211

[bib43] McCusker, D., Denison, C., Anderson, S., Egelhofer, T.A., Yates, J.R., 3rd, Gygi, S.P., and Kellogg, D.R. (2007). Cdk1 coordinates cell-surface growth with the cell cycle. Nat. Cell Biol. 9, 506-515. 10.1038/ncb1568 17417630

[bib44] McGrath, D.A., Balog, E.R.M., Koivomagi, M., Lucena, R., Mai, M.V., Hirschi, A., Kellogg, D.R., Loog, M., and Rubin, S.M. (2013). Cks confers specificity to phosphorylation-dependent CDK signaling pathways. Nat. Struct. Mol. Biol. 20, 1407-1414. 10.1038/nsmb.2707 PMC4242096 24186063

[bib45] McMillan, J.N., Sia, R.A., and Lew, D.J. (1998). A morphogenesis checkpoint monitors the actin cytoskeleton in yeast. J. Cell Biol. 142, 1487-1499. 10.1083/jcb.142.6.1487 PMC2141759 9744879

[bib46] McMillan, J.N., Sia, R.A., Bardes, E.S., and Lew, D.J. (1999). Phosphorylation-independent inhibition of Cdc28p by the tyrosine kinase Swe1p in the morphogenesis checkpoint. Mol. Cell. Biol. 19, 5981-5990. 10.1128/mcb.19.9.5981 PMC84473 10454545

[bib47] Mimura, S., Seki, T., Tanaka, S., and Diffley, J.F.X. (2004). Phosphorylation-dependent binding of mitotic cyclins to Cdc6 contributes to DNA replication control. Nature 431, 1118-1123. 10.1038/nature03024 15496876

[bib48] Morgan, D.O. (2007). The Cell Cycle: Principles of Control (New Science Press).

[bib49] Nguyen, V.Q., Co, C., and Li, J.J. (2001). Cyclin-dependent kinases prevent DNA re-replication through multiple mechanisms. Nature 411, 1068-1073. 10.1038/35082600 11429609

[bib50] Ord, M., and Loog, M. (2019). How the cell cycle clock ticks. Mol. Biol. Cell 30, 169-172. 10.1091/mbc.E18-05-0272 PMC6589557 30640587

[bib51] Pecani, K., and Cross, F.R. (2016). Degradation of the mitotic cyclin Clb3 is not required for mitotic exit but is necessary for G1 cyclin control of the succeeding cell cycle. Genetics 204, 1479-1494. 10.1534/genetics.116.194837 PMC5161280 27794027

[bib52] Perkins, G., Drury, L.S., and Diffley, J.F. (2001). Separate SCF(CDC4) recognition elements target Cdc6 for proteolysis in S phase and mitosis. EMBO J. 20, 4836-4845. 10.1093/emboj/20.17.4836 PMC125267 11532947

[bib53] Petri, E.T., Errico, A., Escobedo, L., Hunt, T., and Basavappa, R. (2007). The crystal structure of human cyclin B. Cell Cycle 6, 1342-1349. 10.4161/cc.6.11.4297 17495533

[bib54] Pettersen, E.F., Goddard, T.D., Huang, C.C., Couch, G.S., Greenblatt, D.M., Meng, E.C., and Ferrin, T.E. (2004). UCSF Chimera--a visualization system for exploratory research and analysis. J. Comput. Chem. 25, 1605-1612. 10.1002/jcc.20084 15264254

[bib55] Phizicky, D.V., Berchowitz, L.E., and Bell, S.P. (2018). Multiple kinases inhibit origin licensing and helicase activation to ensure reductive cell division during meiosis. eLife 7, e33309. 10.7554/eLife.33309 PMC5805409 29388912

[bib56] Puig, O., Caspary, F., Rigaut, G., Rutz, B., Bouveret, E., Bragado-Nilsson, E., Wilm, M., and Seraphin, B. (2001). The tandem affinity purification (TAP) method: a general procedure of protein complex purification. Methods 24, 218-229. 10.1006/meth.2001.1183 11403571

[bib57] Queralt, E., Lehane, C., Novak, B., and Uhlmann, F. (2006). Downregulation of PP2A(Cdc55) phosphatase by separase initiates mitotic exit in budding yeast. Cell 125, 719-732. 10.1016/j.cell.2006.03.038 16713564

[bib58] Rahal, R., and Amon, A. (2008). Mitotic CDKs control the metaphase-anaphase transition and trigger spindle elongation. Genes Dev. 22, 1534-1548. 10.1101/gad.1638308 PMC2418589 18519644

[bib59] Reynard, G.J., Reynolds, W., Verma, R., and Deshaies, R.J. (2000). Cks1 is required for G(1) cyclin-cyclin-dependent kinase activity in budding yeast. Mol. Cell. Biol. 20, 5858-5864. 10.1128/mcb.20.16.5858-5864.2000 PMC86063 10913169

[bib60] Schulman, B. A., Lindstrom, D. L., and Harlow, E. D. (1998). Substrate recruitment to cyclin-dependent kinase 2 by a multipurpose docking site on cyclin A. Proc. Natl. Acad. Sci. U.S.A. 95, 10453-10458. 10.1073/pnas.95.18.10453 PMC27915 9724724

[bib61] Segal, M., Bloom, K., and Reed, S. I. (2000). Bud6 Directs Sequential Microtubule Interactions with the Bud Tip and Bud Neck during Spindle Morphogenesis in Saccharomyces cerevisiae. Mol. Biol. Cell 11, 3689-3702. 10.1091/mbc.11.11.3689 PMC15030 11071900

[bib62] Stern, B., and Nurse, P. (1996). A quantitative model for the cdc2 control of S phase and mitosis in fission yeast. Trends Genet. 12, 345-350. 8855663

[bib63] Swaffer, M.P., Jones, A.W., Flynn, H.R., Snijders, A.P., and Nurse, P. (2016). CDK substrate phosphorylation and ordering the cell cycle. Cell 167, 1750-1761.e16. 10.1016/j.cell.2016.11.034 PMC5161751 27984725

[bib64] Tanaka, S., Umemori, T., Hirai, K., Muramatsu, S., Kamimura, Y., and Araki, H. (2007). CDK-dependent phosphorylation of Sld2 and Sld3 initiates DNA replication in budding yeast. Nature 445, 328-332. 10.1038/nature05465 17167415

[bib65] Tomson, B.N., Rahal, R., Reiser, V., Monje-Casas, F., Mekhail, K., Moazed, D., and Amon, A. (2009). Regulation of Spo12 phosphorylation and its essential role in the FEAR network. Curr. Biol. 19, 449-460. 10.1016/j.cub.2009.02.024 PMC2692463 19268588

[bib66] Topacio, B.R., Zatulovskiy, E., Cristea, S., Xie, S., Tambo, C.S., Rubin, S.M., Sage, J., Koivomagi, M., and Skotheim, J.M. (2019). Cyclin D-Cdk4,6 drives cell-cycle progression via the retinoblastoma protein’s C-terminal helix. Mol. Cell. Published online April 11, 2019. 10.1016/j.molcel.2019.03.020 10.1016/j.molcel.2019.03.020 PMC6800134 30982746

[bib67] Ubersax, J.A., Woodbury, E.L., Quang, P.N., Paraz, M., Blethrow, J.D., Shah, K., Shokat, K.M., and Morgan, D.O. (2003). Targets of the cyclin-dependent kinase Cdk1. Nature 425, 859-864. 10.1038/nature02062 14574415

[bib68] Visintin, R., Prinz, S., and Amon, A. (1997). CDC20 and CDH1: a family of substrate-specific activators of APC-dependent proteolysis. Science 278, 460-463. 10.1126/science.278.5337.460 9334304

[bib69] Wasch, R., and Cross, F.R. (2002). APC-dependent proteolysis of the mitotic cyclin Clb2 is essential for mitotic exit. Nature 418, 556-562. 10.1038/nature00856 12152084

[bib70] Williams, B.C., Filter, J.J., Blake-Hodek, K.A., Wadzinski, B.E., Fuda, N.J., Shalloway, D., and Goldberg, M.L. (2014). Greatwall-phosphorylated Endosulfine is both an inhibitor and a substrate of PP2A-B55 heterotrimers. eLife 3, e01695. 10.7554/eLife.01695 PMC3949306 24618897

[bib71] Wilmes, G.M., Archambault, V., Austin, R.J., Jacobson, M.D., Bell, S.P., and Cross, F.R. (2004). Interaction of the S-phase cyclin Clb5 with an “RXL” docking sequence in the initiator protein Orc6 provides an origin-localized replication control switch. Genes Dev. 18, 981-991. 10.1101/gad.1202304 PMC406289 15105375

[bib72] Zegerman, P., and Diffley, J.F.X. (2007). Phosphorylation of Sld2 and Sld3 by cyclin-dependent kinases promotes DNA replication in budding yeast. Nature 445, 281-285. 10.1038/nature05432 17167417

