# Supplementary material for: Cyclin-Specific Docking Mechanisms Reveal the Complexity of M-CDK Function in the Cell Cycle
Source: Mol Cell. 2019 Jul 11;75(1):76–89.e3. doi: 10.1016/j.molcel.2019.04.026 (PMC6620034; doi:10.1016/j.molcel.2019.04.026)
Supplement: Document S1. Figures S1–S7 and Tables S1–S4 [file mmc1.pdf]

Molecular Cell, Volume *75*

Supplemental Information

Cyclin-Specific Docking Mechanisms Reveal the Complexity of M-CDK Function in the Cell Cycle

Mihkel Örd, Rainis Venta, Kaidi Möll, Ervin Valk, and Mart Loog

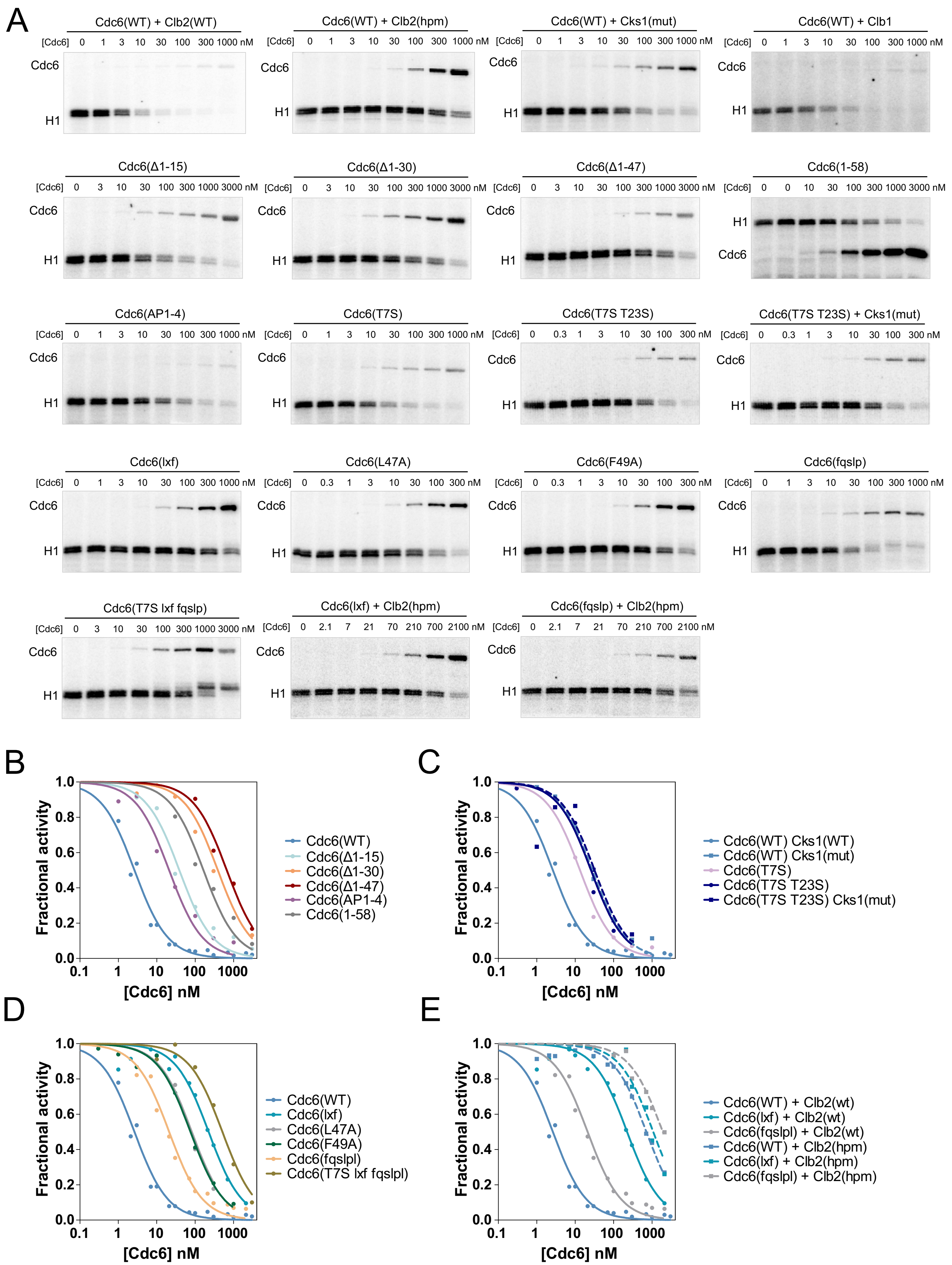

Figure S1. Analysis of the interactions that lead to inhibition of M-CDK by Cdc6, Related to Figure 1. (A) Autoradiographs that show the inhibition assays used to determine the K_i values for Cdc6 mutants (see tables in Figure 1E-G). Kinase assays were performed with Clb2(WT)-Cdk1-Cks1(WT) unless noted otherwise. (B-E) The inhibition curves obtained from non-linear regression fits of the data from the experiments presented in 'A'.

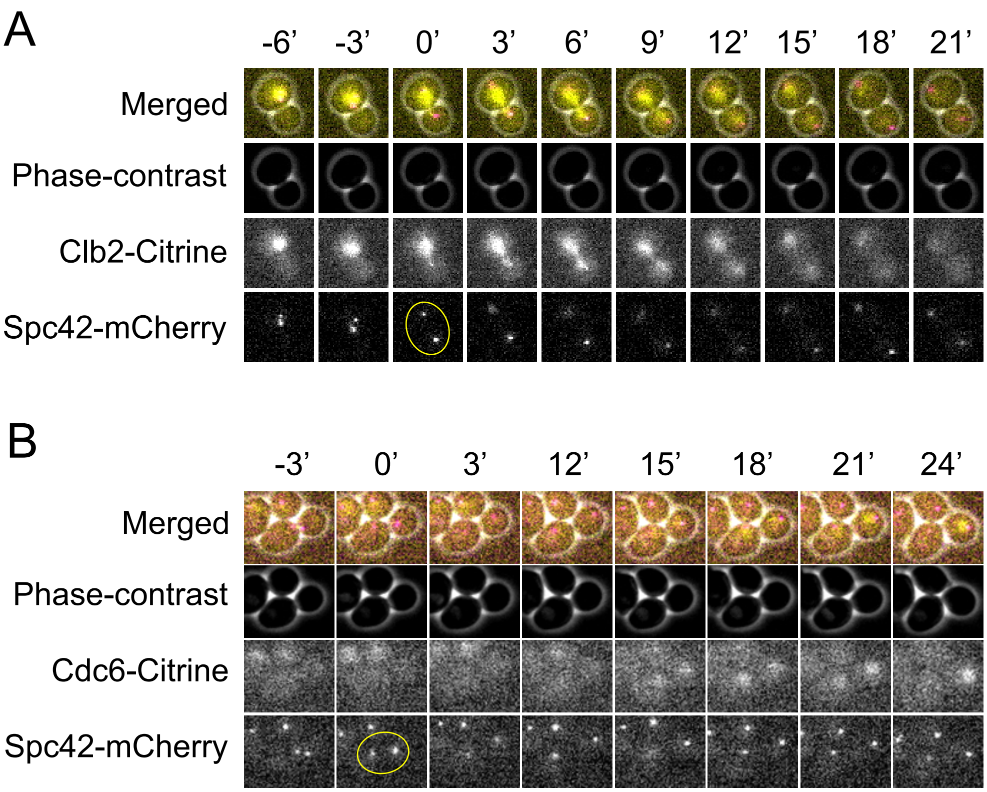

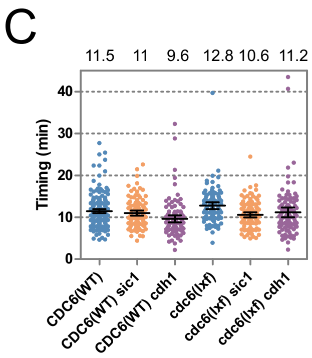

Figure S2. The levels of Clb2, Cdc6 and nuclear accumulation of the NLS-NES-GFP Cdk1 activity sensor in mitotic exit, Related to Figure 2. (A) Live cell microscopy images showing the degradation of Clb2-Citrine during mitotic exit. SPBs are tagged by Spc42-mCherry. The yellow oval marks the onset of spindle elongation. (B) Microscopy images of cells expressing Cdc6-Citrine and Spc42-mCherry. The onset of anaphase is marked by a yellow oval. (C) The timings of NLS-NES-GFP Cdk1 activity sensor reaching 50% of its G1 nuclear level after spindle elongation in indicated strains. The numbers above the plot show the average for each strain. Error bars show 95% confidence intervals of the mean.

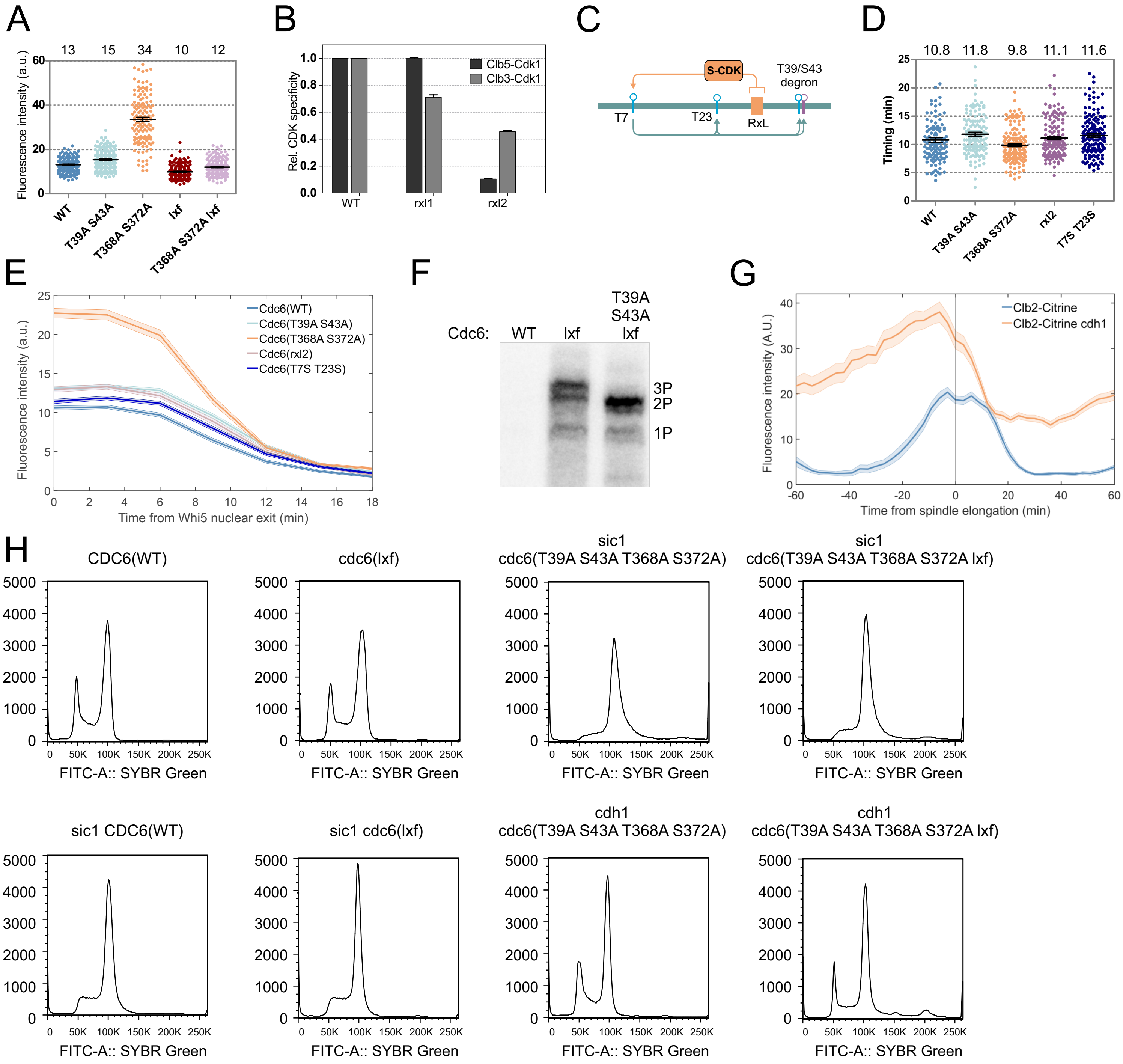

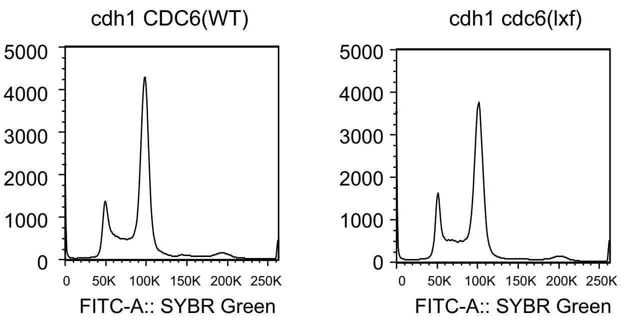

Figure S3. Regulation of Cdc6 stability and the effect of Cdc6 LxF motif on cell cycle progression and re-replication, Related to Figure 3. (A) Cdc6-Citrine accumulation during mitotic exit was studied in live cell microscopy experiments. This plot shows maximum Cdc6-Citrine intensities during the G1-phase of individual cells. The numbers above the plot show the average for each strain. (B) The influence of RxL motif mutations (rxl1 (^{14}ANAF), rxl2 (^{31}AKAQF)) on Cdc6 phosphorylation by Clb5- or Clb3-Cdk1 in a kinase assay. Relative CDK specificity shows the initial rate of phosphorylation for the indicated Cdc6 mutants compared to the wild type Cdc6. (C) Scheme showing S-CDK *hp*-RxL and Cks1-mediated docking interactions in phosphorylation of Cdc6 N-terminal domain. (D) Cdc6-Citrine degradation after *Start* was examined in time-lapse microscopy experiments using nuclear export of Whi5-mCherry as an indicator for *Start*. This plot shows the nuclear intensity of Cdc6-Citrine variants averaged over a population of cells. (E) Timing of Cdc6-Citrine degradation after *Start* indicated by the time from *Start* to degradation of 50% of Cdc6 in individual cells. The numbers above the plot indicate the mean timing values for Cdc6 variants. (F) Cdc6 variants were phosphorylated with Clb2-Cdk1, an autoradiograph of the reactions separated on Phos-tag SDS-PAGE is shown. (G) Levels of Clb2-Citrine 60 minutes before and after the metaphase-anaphase transition in wild type or *cdh1* deletion cells. The fluorescence intensities are averaged over a population of cells synchronized at the point of spindle elongation. (H) Flow cytometry profiles of asynchronous cultures for the indicated strains stained with SYBR Green.

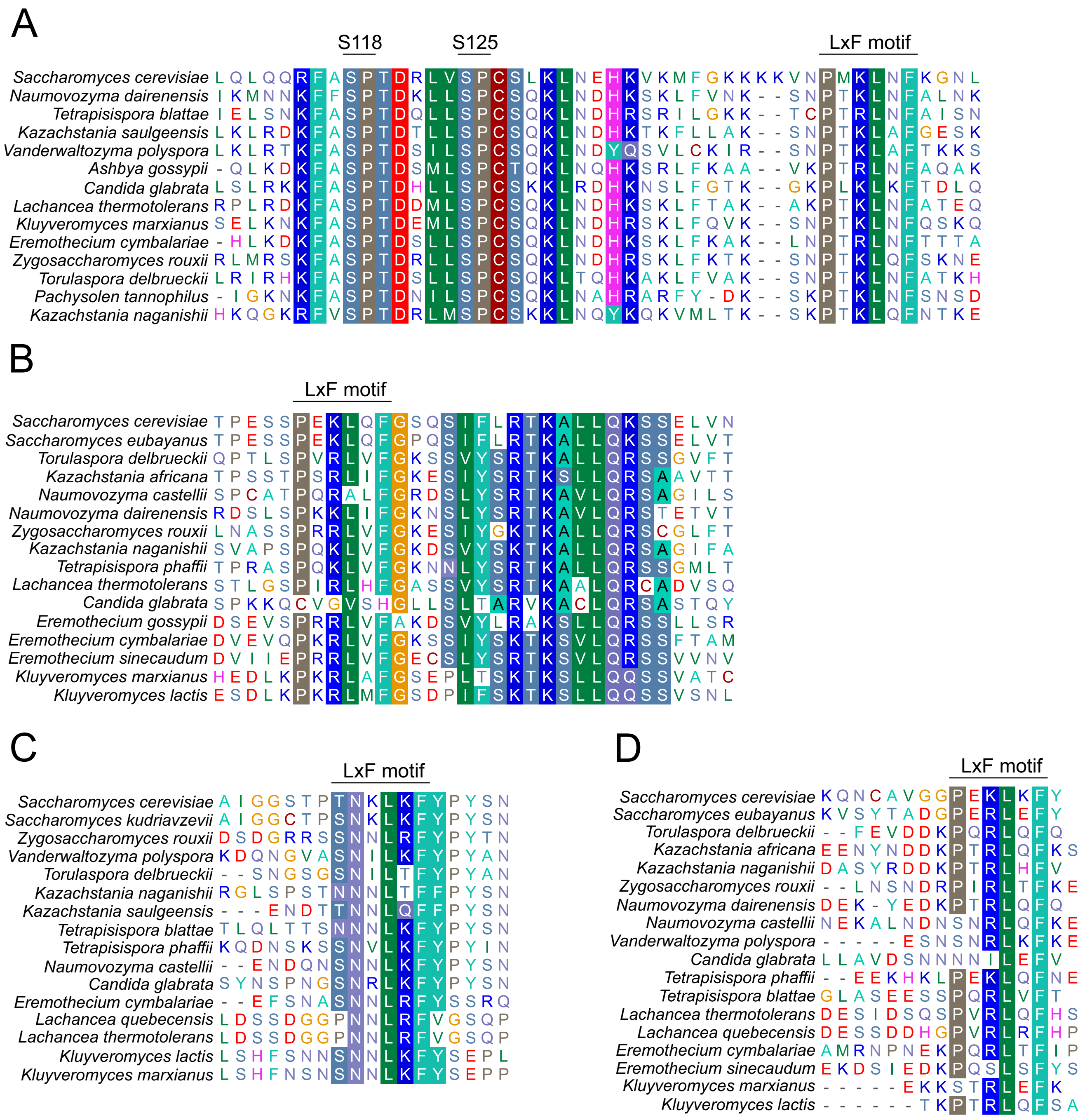

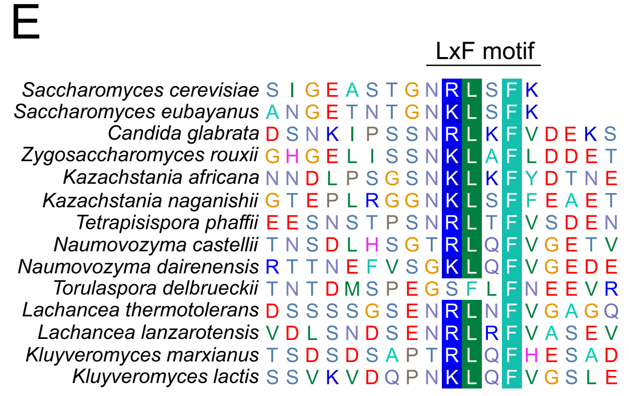

Figure S4. Conservation of the experimentally verified LxF motifs in Spo12, Cdc6, Swe1, Bni1, and Bud3, Related to Figure 4. Sequence alignments of the functional LxF motifs in these proteins confirm the central importance of the leucine and phenylalanine in the motif and shows conservation of residues in positions -3, -2 and -1 from the leucine. The figure shows sequence alignments of LxF motifs in Spo12 (A), Cdc6 (B), Swe1 (C), Bud3 (D) and Bni1 (E).

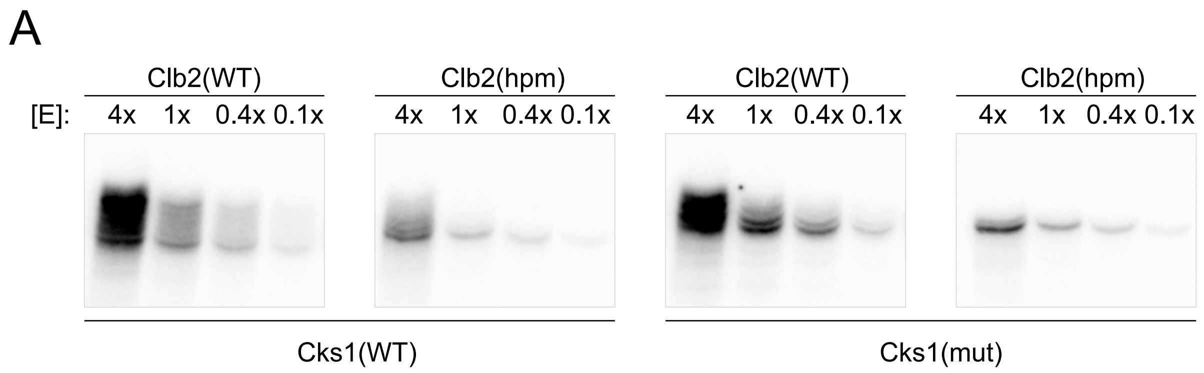

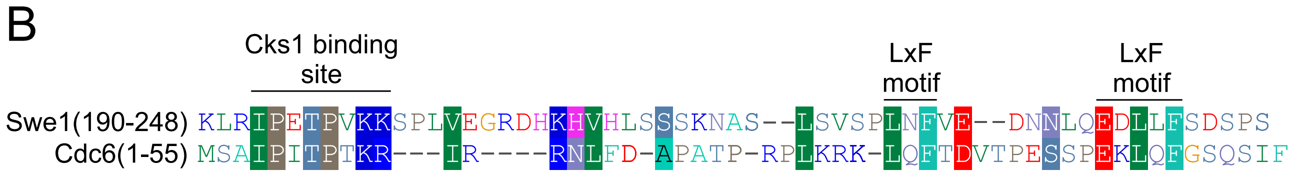

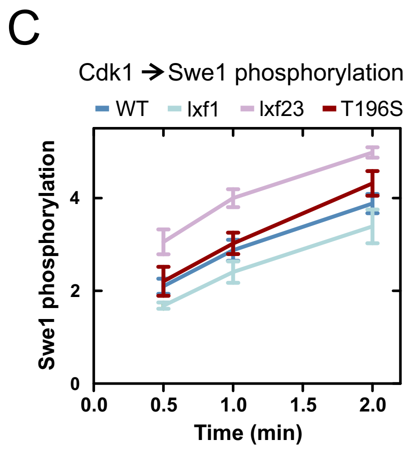

Figure S5. Clb2-Cdk1 dependent phosphorylation of Swe1, Related to Figure 5. (A) Autoradiographs of phosphorylation reactions containing the N-terminal domain of Swe1 as a substrate for either the wild type or *hp* mutant (Clb2(hpm)) M-CDK complex in the presence of either the wild type Cks1 or Cks1(mut) (phospho-pocket mutant). Autoradiography scans of phosphorylated forms separated using Phos-tag SDS-PAGE are shown. (B) Alignment of Cdc6(1-55) and Swe1(190-248) reveals similar positioning of Cks1 binding threonine-based priming phosphorylation site and LxF motifs. (C) Quantified profiles of Swe1 phosphorylation in the two-way assay containing Swe1 and Clb2-Cdk1. The error bars show standard deviation of the mean.

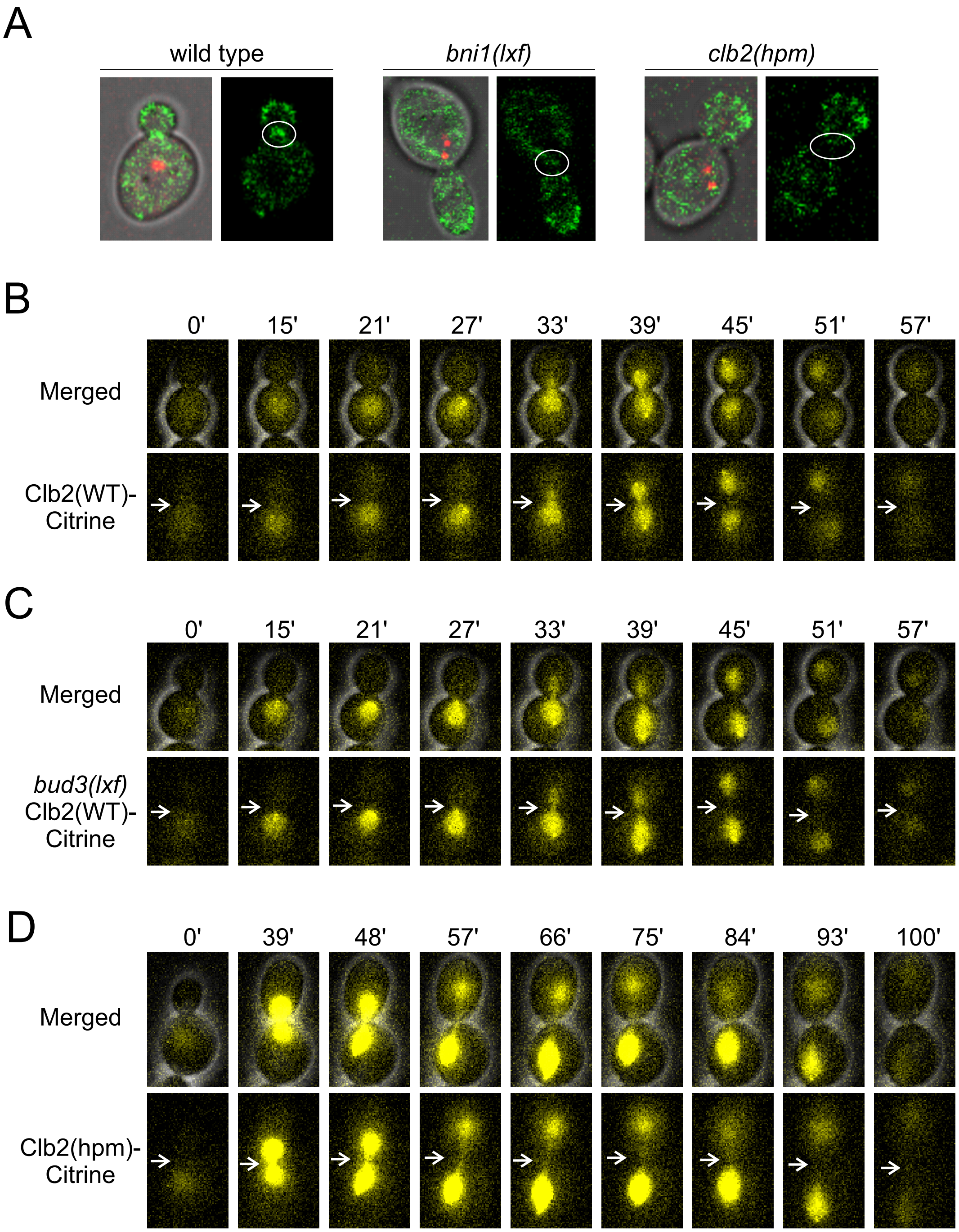

Figure S6. The LxF mediates bud neck localization of Clb2 and Bud6, Related to Figure 6. (A) Microscopy images showing localization of GFP-Bud6 in wild type, *bni1(lxf)* or clb2(hpm) metaphase cells. SPBs are tagged by Spc42-mCherry. The images on the left are merged from brightfield, GFP and mCherry channels. The images on the right show GFP-Bud6 signals. The white oval marks the bud neck. (B-D) The localization of Clb2-Citrine was studied using time-lapse microscopy. The images show expression and localization of Clb2-Citrine in wild type cell (B), bud3(lxf) cell (C) and clb2(hpm) cell (D).

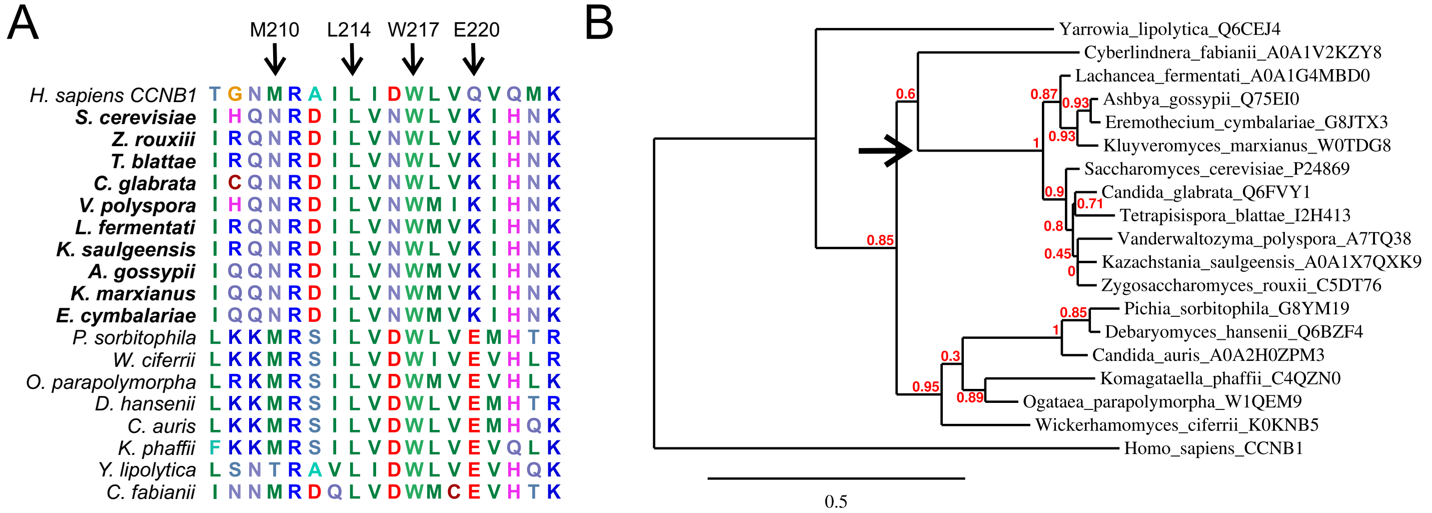

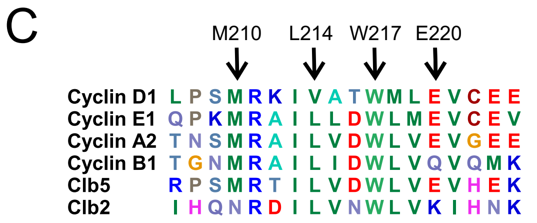

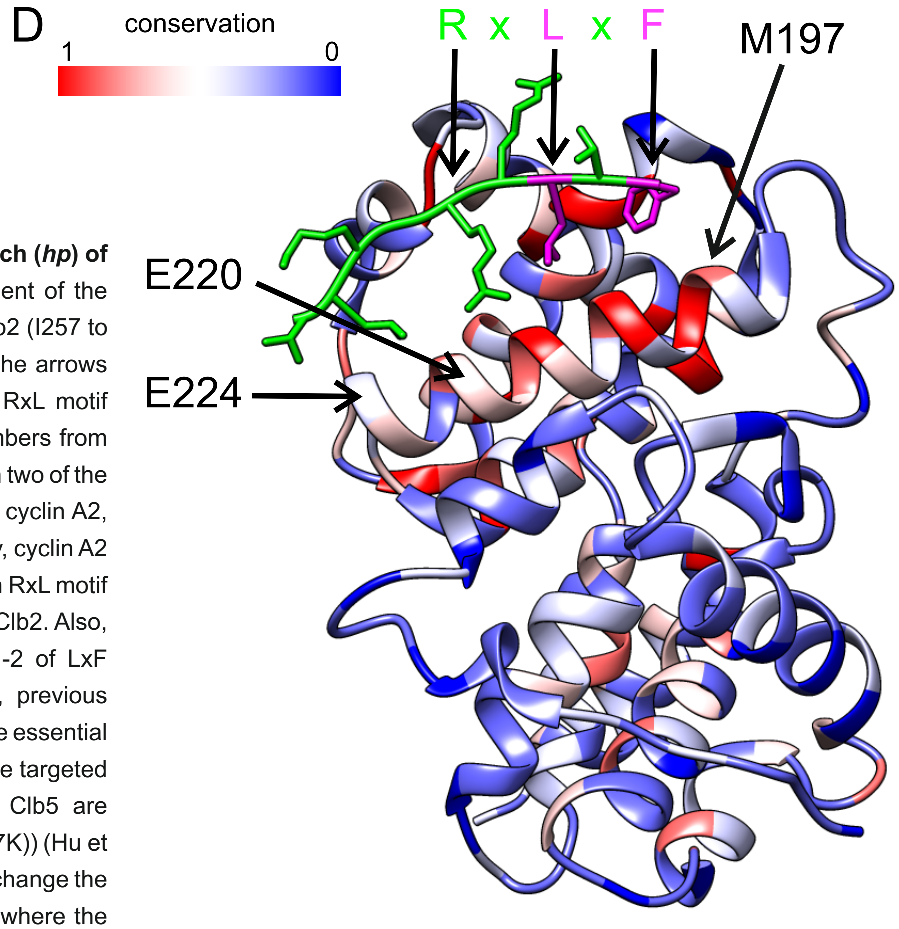

Figure S7. Conservation of the hydrophobic patch (*hp*) of Clb2, Related to Figure 6. (A) Sequence alignment of the hydrophobic patches of human cyclin B1 and of Clb2 (I257 to K274) and its homologues from budding yeasts. The arrows above indicate the critical amino acids in *hp* for RxL motif binding to cyclin A2 (the numbers are residue numbers from cyclin A2) (Schulman et al., 1998). Differences are in two of the indicated positions: in positions M210 and E220 of cyclin A2, Clb2 has N260 and K270, respectively. Interestingly, cyclin A2 E220 interacts with the positively charged residue in RxL motif (Schulman et al., 1998) and is mutated to lysine in Clb2. Also, many of the LxF motifs have E/N/Q in position -2 of LxF (compared to R/KxLxF) (Figure 6A). Importantly, previous reports have shown that the Clb2 N260 and K270 are essential for Swe1 regulation of M-CDK and that Swe1 can be targeted to inhibit Clb5-Cdk1 when the two positions in Clb5 are mutated to N and K, respecively (Clb5(M197N E207K)) (Hu et al., 2008). This indicates that these positions could change the binding specificity of target peptides. The species where the hydrophobic patch is conserved with Clb2 are in bold.
(B) A phylogenetic tree constructed from sequence alignment of the B-type cyclins of panel 'A'. The arrow points to the clade where the critical hydrophobic patch residues are conserved with *S. cerevisiae* Clb2. (C) Alignment of the hydrophobic patch sequences of human cyclins D1, E1, A2, B1 and *S. cerevisiae* cyclins Clb5 and Clb2. The arrows point to RxL-binding residues in cyclin A2 (Schulman 1998). Cyclin A2 position E220 interacts with the R/K in RxL motif and is glutamate in all S-phase cyclins (E1, A2 and Clb5), but is mutated in mitotic cyclins (glutamine in cyclin B1 and lysine in Clb2). Cyclin B1 shows weak affinity to the canonical RxL motif and, based on structural studies, a mutation in this position (Q211 in cyclin B1) is expected to weaken the RxL interaction (Brown et al., 2007; Petri et al., 2007). The hydrophobic patch is not identical in Clb2 and cyclin B1, but both carry a mutation in the position E220 of cyclin A2, which suggests that mammalian cyclin B could have a specific docking motif similarly to Clb2. (D) Structure of human cyclin A2 bound with RxLxF peptide (TLKGRRLVF) from 2CCI (Cheng et al., 2006). The substrate peptide containing the RxL motif is in green, with L and F in magenta. The residues in cyclin structure are coloured based on conservation in the cyclins aligned in 'C'. Red indicates high conservation and blue high variation. The residues surrounding the area where LxF in the substrate peptide is bound show very high conservation, whereas there is much more variation in the C-terminal side of cyclin α1 helix, which interacts with the residues N-terminal of the LxF in the substrate peptide. This suggests that the hydrophobic core (LxF, LxΦ or LΦ, where Φ is M/L/F/P) of the cyclin docking motifs could be similar for different cyclins, but greater differences might arise from the residues surrounding the hydrophobic interaction core. Further biochemical studies are necessary to determine the cyclin specificity of various target peptide motifs in metazoans.

Table S1. List of predicted LxF motifs in disordered regions of *S.cerevisiae* proteome, Related to Figure 6.

GeneName	Hit	SeqStart	SeqStop	IUPred
ADA2	vqgfmPGRLEFetefe	167	172	0.517
ALA1	gslvaPEKLRFdfshk	652	657	0.305
ALT2	elkknPEVLPFddiin	61	66	0.326
AQR1	gqtksEKLNFegayi	37	41	0.554
BNI1	eastgNRLSFk	1948	1952	0.767
BUD3	cavggPEKLKFy	1630	1635	0.611
CDC6	tpessPEKLQFgsqsi	44	49	0.449
CEM1	ensaiNKLLFtsqde	75	79	0.378
CLU1	dvtdeEKLKFnemvh	182	186	0.352
DBP10	kneekEKLDFlaklq	671	675	0.342
DNF2	ntqfcPEDLTFvskei	786	791	0.395
FIN1	ifptsPTKLTFsnenk	118	123	0.4
FMT1	cgalsEKLVFl	396	400	0.334
GCV3	nalnkNKLPFlyssq	31	35	0.401
GLC3	nefghPEWLDFpnvnn	551	556	0.305
HRF393/HRD1054	lpknaPQKLNFremrq	514	519	0.337
HRQ1	tkrkrPARLIFydskg	940	945	0.387
IES6	ndggdERLLFlrsvg	28	32	0.525
ISA1	innvnPFKLKFipktv	41	46	0.323
LPZ3C	mivriPEPLTFtnsed	590	595	0.342
LTE1	tdengPQRLLFhetdk	594	599	0.688
MCM3	vssrhPWKLSFkgsfg	152	157	0.303
MFB1	kaelfEKLIFrdsrp	292	296	0.32
MGA2	degdsPNLLSFegign	71	76	0.52
MRP51	ggtqwNRLRFqemgv	86	90	0.345
N1400	hnqakERLLFlqrle	518	522	0.308
NAM9	msdsnPKKLQFqeflr	212	217	0.409
NCS6	klralEKLSF	355	359	0.51
NSR1	yidnrPVRLDFssprp	337	342	0.584
PEX13	sepidPSKLEFaraly	305	310	0.37
RAD53	qtskgPENLQFs	815	820	0.855
RKM3	sivneEKLPFlakkd	485	489	0.323
RRP12	vrgqrNKLKFrkngk	1175	1179	0.591
SAM2	yswekPKKLEF	379	384	0.643
SCC2	neaekERLVFkrpsn	82	86	0.408
SCS22	mrivPEKLVFkapln	5	10	0.42
SEG1	npsmtNRLRFssnpe	917	921	0.702
SES1	yipgePEFLPFvnelp	439	444	0.305
SMC1	iltveNKLQFetdrl	830	834	0.326

SPC42	ilpinNRLNFqepkr	227	231	0.464
SPO12	kkkvnPMKLNFkgnla	147	152	0.343
SWD2	vsinkPNLLKFkhvks	9	14	0.307
SWE1	gstptNKLKFypysn	48	52	0.381
TAF11	sinkvPENLIFpqdil	86	91	0.565
TAF4	nnvkiPNHLPFlhpeq	181	186	0.478
TDA11	skrssNKLSFigepd	480	484	0.627
TRP1	grqesPESLHFmlagg	170	175	0.336
TUS1	srersPNKLSFignse	136	141	0.637
UBC7	dyplsPPKLTFtpsil	68	73	0.322
UBP13	nepgaPSRLSFenvtd	291	296	0.671
UBP15	sissnEKLTFyeevq	816	820	0.316
UTP21	apkksEKLPFflqls	754	758	0.493
VMA1	ssrevPELLKFtcnat	351	356	0.391
VPS36	vrsklNRLNFhdsrv	215	219	0.376
VTC3	idsniPNPLRFlrage	473	478	0.33
YEL077C	lpknaPQKLNFremrq	68	73	0.337
YHP1	pkgkeNRLKFnayer	331	335	0.485
YIL177C	lpknaPQKLNFremrq	514	519	0.337
YIR016W	vpaeqEKLSFlakas	160	164	0.3
YKR075C	kkeqfPRKLKFnqavm	222	227	0.425
YMR135W-A	nsntmERLPFtrngs	120	124	0.581
YOR385W	qrntiPERLHFsrern	26	31	0.537
YRF1-1	lpknaPQKLNFremrq	643	648	0.337
YRF1-2	lpknaPQKLNFremrq	529	534	0.337
YRF1-3	lpknaPQKLNFremrq	707	712	0.337
YRF1-4	lpknaPQKLNFremrq	229	234	0.337
YRF1-5	lpknaPQKLNFremrq	643	648	0.337
YRF1-6	lpknaPQKLNFremrq	707	712	0.337
YRF1-7	lpknaPQKLNFremrq	707	712	0.337
YRF1-8	lpknaPQKLNFremrq	643	648	0.337
ZDS1	tsilpPRKLTFadvkk	808	813	0.523
ZDS2	istlpPRKLTFedvvk	819	824	0.444

Table S2. Yeast strains used in this study, Related to STAR Methods. All yeast strains are based on MATa derivate of strain w303 unless noted otherwise.

Name	Genotype
DMY305	cln2::TRP1::GAL1-3HA-CLN2
DOM0076	bar1Δ::HISG sic1Δ::LEU2 pRS426-GAL1-CLB5-TAP
DOM0077	bar1Δ::HISG sic1Δ::LEU2 pRS426-GAL1-CLB2-TAP
DOM0957	bar1Δ::HISG sic1Δ::LEU2 pRS426-GAL1-CLB3-TAP
DOM0963	bar1Δ::HISG sic1Δ::LEU2 pRS426-GAL1-CLB5hpm-TAP
DOM0964	bar1Δ::HISG sic1Δ::LEU2 pRS426-GAL1-CLB2hpm-TAP
MK0168	bar1Δ::HISG sic1Δ::LEU2 pRS426-GAL1-CLB3hpm-TAP
PPY2444	bar1Δ cln2::TRP1::GAL1-3HA-cln2(lpd)
MO251	bar1Δ::HISG spc42::SPC42-mCherry::kanMX4 clb2::CLB2-yeCitrine::HIS3
MO256	bar1Δ::HISG spc42::SPC42-mCherry::kanMX4 cdc6::natNT2::GALS-3HA-CDC6 ura3::P_{CDC6}-CDC6-yeCitrine::URA3
MO235	bar1Δ::HISG whi5::WHI5-mCherry::SpHIS5 cdc6::natNT2::GALS-3HA-CDC6 ura3::P_{CDC6}-CDC6-yeCitrine::URA3
MO236	bar1Δ::HISG whi5::WHI5-mCherry::SpHIS5 cdc6::natNT2::GALS-3HA-CDC6 ura3::P_{CDC6}-CDC6(rxl2)-yeCitrine::URA3
MO237	bar1Δ::HISG whi5::WHI5-mCherry::SpHIS5 cdc6::natNT2::GALS-3HA-CDC6 ura3::P_{CDC6}-CDC6(T39A S43A)-yeCitrine::URA3
MO238	bar1Δ::HISG whi5::WHI5-mCherry::SpHIS5 cdc6::natNT2::GALS-3HA-CDC6 ura3::P_{CDC6}-CDC6(T7S T23S)-yeCitrine::URA3
MO258	bar1Δ::HISG whi5::WHI5-mCherry::SpHIS5 cdc6::natNT2::GALS-3HA-CDC6 ura3::P_{CDC6}-CDC6(T368A S372A)-yeCitrine::URA3
MO184	bar1Δ::HISG whi5::WHI5-mCherry::SpHIS5 cdc6::natNT2::GALS-3HA-CDC6 ura3::P_{CDC6}-CDC6(lxf T368A S372A)-yeCitrine::URA3
MO268	bar1Δ::HISG whi5::WHI5-mCherry::SpHIS5 cdc6::natNT2::GALS-3HA-CDC6 ura3::P_{CDC6}-CDC6(lxf)-yeCitrine::URA3
MO302	bar1Δ::HISG whi5::WHI5-mCherry::SpHIS5 cdc6::natNT2::GALS-3HA-CDC6 ura3::P_{CDC6}-CDC6-yeCitrine::URA3 cdh1Δ::LEU2
MO303	bar1Δ::HISG whi5::WHI5-mCherry::SpHIS5 cdc6::natNT2::GALS-3HA-CDC6 ura3::P_{CDC6}-CDC6(lxf T368A S372A)-yeCitrine::URA3 cdh1Δ::LEU2
MO304	bar1Δ::HISG whi5::WHI5-mCherry::SpHIS5 cdc6::natNT2::GALS-3HA-CDC6 ura3::P_{CDC6}-CDC6(T368A S372A)-yeCitrine::URA3 cdh1Δ::LEU2
MO115	bar1Δ::HISG spc42::SPC42-mCherry::kanMX4 P_{ADH1}::TRP1::P_{ADH1}-(NLS-NES-GFP)
MO116	bar1Δ::HISG spc42::SPC42-mCherry::kanMX4 P_{ADH1}::TRP1::P_{ADH1}-(NLS-NES-GFP) clb2::clb2(hpm)
MO155	bar1Δ::HISG spc42::SPC42-mCherry::kanMX4 P_{ADH1}::TRP1::P_{ADH1}-(NLS-NES-GFP) clb2::clb2(hpm) ura3::URA3:: P_{CLB2}-CLB2
MO227	bar1Δ::HISG spc42::SPC42-mCherry::kanMX4 P_{ADH1}::TRP1::P_{ADH1}-(NLS-NES-GFP) spo12 Δ::URA3
MO281	bar1Δ::HISG spc42::SPC42-mCherry::kanMX4 P_{ADH1}::TRP1::P_{ADH1}-(NLS-NES-GFP) spo12::spo12(RxL)

MO232	bar1Δ::HISG spc42::SPC42-mCherry::kanMX4 P_{ADH1}::TRP1::P_{ADH1}-(NLS-NES-GFP) spo12::spo12(lxf)-13MYC::HIS3
MO313	bar1Δ::HISG spc42::SPC42-mCherry::kanMX4 P_{ADH1}::TRP1::P_{ADH1}-(NLS-NES-GFP) spo12::SPO12-13MYC::HIS3
MO248	bar1Δ::HISG spc42::SPC42-mCherry::kanMX4 P_{ADH1}::TRP1::P_{ADH1}-(NLS-NES-GFP) cdc6::natNT2::GALS-3HA-CDC6 ura3::P_{CDC6}-CDC6-6HA::URA3
MO249	bar1Δ::HISG spc42::SPC42-mCherry::kanMX4 P_{ADH1}::TRP1::P_{ADH1}-(NLS-NES-GFP) cdc6::natNT2::GALS-3HA-CDC6 ura3::P_{CDC6}-CDC6-6HA::URA3 cdh1Δ::HIS3
MO250	bar1Δ::HISG spc42::SPC42-mCherry::kanMX4 P_{ADH1}::TRP1::P_{ADH1}-(NLS-NES-GFP) cdc6::natNT2::GALS-3HA-CDC6 ura3::P_{CDC6}-CDC6-6HA::URA3 sic1Δ::LEU2
MO253	bar1Δ::HISG spc42::SPC42-mCherry::kanMX4 P_{ADH1}::TRP1::P_{ADH1}-(NLS-NES-GFP) cdc6::natNT2::GALS-3HA-CDC6 ura3::P_{CDC6}-CDC6(lxf)-6HA::URA3
MO254	bar1Δ::HISG spc42::SPC42-mCherry::kanMX4 P_{ADH1}::TRP1::P_{ADH1}-(NLS-NES-GFP) cdc6::natNT2::GALS-3HA-CDC6 ura3::P_{CDC6}-CDC6(lxf)-6HA::URA3 sic1Δ::LEU2
MO255	bar1Δ::HISG spc42::SPC42-mCherry::kanMX4 P_{ADH1}::TRP1::P_{ADH1}-(NLS-NES-GFP) cdc6::natNT2::GALS-3HA-CDC6 ura3::P_{CDC6}-CDC6(lxf)-6HA::URA3 cdh1Δ::HIS3
MO343	BY4741 spc42::SPC42-mCherry::kanMX4
MO347	BY4741 spc42::SPC42-mCherry::kanMX4 swe1Δ::URA3
MO349	BY4741 spc42::SPC42-mCherry::kanMX4 swe1::swe1(lxf23)
MO356	BY4741 spc42::SPC42-mCherry::kanMX4 swe1::swe1(T196S)
MO360	BY4741 spc42::SPC42-mCherry::kanMX4 swe1::swe1(lxf1)
MO222	bar1Δ::HISG clb2::CLB2-yeCitrine::HIS3
MO234	bar1Δ::HISG clb2::CLB2-yeCitrine::HIS3 bud3::bud3(lxf)
MO247	bar1Δ::HISG clb2::clb2(hpm)-yeCitrine::HIS3
MO340	bar1Δ::HISG spc42::SPC42-mCherry::kanMX4 bud6::natNT2::P_{CYC1}-EGFP-BUD6
MO341	bar1Δ::HISG spc42::SPC42-mCherry::kanMX4 bud6::natNT2::P_{CYC1}-EGFP-BUD6 clb2::clb2(hpm)
MO342	bar1Δ::HISG spc42::SPC42-mCherry::kanMX4 bud6::natNT2::P_{CYC1}-EGFP-BUD6 bni1::bni1(lxf)
MO261	bar1Δ::HISG spc42::SPC42-mCherry::kanMX4 clb2::CLB2-yeCitrine::HIS3 cdh1Δ::URA3
MO474	bar1Δ::HISG whi5::WHI5-mCherry::SpHIS5 cdc6::natNT2::GALS-3HA-CDC6 ura3::P_{CDC6}-CDC6(T39A S43A T368A S372A lxf)-yeCitrine::URA3

Table S3. List of plasmids used in the study, Related to STAR Methods.

Plasmid number	Description
pRV1	6xHis-Cdc6 pET28a
pMO268	6xHis-Cdc6(d1-15) pET28a
pMO269	6xHis-Cdc6(d1-30) pET28a
pMO257	6xHis-Cdc6(d1-47) pET28a
pRV45	6xHis-Cdc6(T7A T23A T39A S43A) pET28a
pMO064	6xHis-Cdc6(1-58)-GB1 pET28a
pRV195	6xHis-Cdc6(T7S) pET28a
pMO300	6xHis-Cdc6(T7S T23S) pET28a
pMO434	6xHis-Cdc6(L47A F49A) pET28a
pMO364	6xHis-Cdc6(L47A) pET28a
pMO356	6xHis-Cdc6(F49A) pET28a
pMO034	6xHis-Cdc6(F126A L129A P130A) pET28a
pMO410	6xHis-Cdc6(T7S L47A F49A F126A L129A P130A) pET28a
pRV2	6xHis-Cdc6(AP T7) pET28a
pRV3	6xHis-Cdc6(AP T23) pET28a
pRV5	6xHis-Cdc6(AP T39) pET28a
pRV7	6xHis-Cdc6(AP S43) pET28a
pMO490	GST-TEV-Spo12 pGEX-4T1
pMO491	GST-TEV-Spo12(P147A L150A F152A) pGEX-4T1
pMO459	Sic1(1-33 T2A T5S+3A)-GB1-6xHis pET28a
pMO460	Sic1(1-26 T2A T5S+3A + PEKLQF)-GB1-6xHis pET28a
pMO471	Sic1(1-26 T2A T5S+3A + AEKLQF)-GB1-6xHis pET28a
pMO472	Sic1(1-26 T2A T5S+3A + PAKLQF)-GB1-6xHis pET28a
pMO473	Sic1(1-26 T2A T5S+3A + PEALQF)-GB1-6xHis pET28a
pMO474	Sic1(1-26 T2A T5S+3A + PEKAQF)-GB1-6xHis pET28a
pMO475	Sic1(1-26 T2A T5S+3A + PEKLAF)-GB1-6xHis pET28a
pMO476	Sic1(1-26 T2A T5S+3A + PEKLQA)-GB1-6xHis pET28a
pMO415	6xHis-Swe1(1-450) pET28a
pMO416	6xHis-Swe1(1-450 E239A L241A F243A) pET28a
pMO455	6xHis-Swe1(1-450 L50A F52A) pET28a
pMO456	6xHis-Swe1(1-450 L229A F231A) pET28a
pMO534	6xHis-Swe1(1-450 L229A F231A E239A L241A F243A) pET28a
pMO560	6xHis-Swe1(1-450 T196S) pET28a
pMO466	P_{GAL1}-Swe1-TAP pRS426
pMO467	P_{GAL1}-Swe1(L50A F52A)-TAP pRS426
pMO529	P_{GAL1}-Swe1(T196S)-TAP pRS426
pMO536	P_{GAL1}-Swe1(L229A F231A E239A L241A F243A)-TAP pRS426
pMO344	GST-Bud3(1222-1636) pGEX-4T1
pMO492	GST-Bni1(1215-1953) pGEX-4T1
pMO357	GST-Bud3(1222-1629) pGEX-4T1
pMO493	GST-Bni1(1215-1953 L1950A F1952A) pGEX-4T1

pMK516	6xHis-Fir1 pET28a
pMO478	6xHis-Fir1(L385A F387A F388A) pET28a
pMO511	Sic1(1-26 T2A T5S+3A +VNRILFP)-GB1-6xHis pET28a
pMO512	Sic1(1-33 T2A T5S+3R)-GB1-6xHis pET28a
pMO513	Sic1(1-26 T2A T5S+3R +PEKLQF)-GB1-6xHis pET28a
pMO514	Sic1(1-26 T2A T5S+3R +VNRILFP)-GB1-6xHis pET28a
pRV53	6xHis-Cdc6(rxl1) pET28a
pRV57	6xHis-Cdc6(rxl2) pET28a
pMO659	6xHis-Cdc6(T39A S43A L47A F49A) pET28a

Table S4. Number of cells used for analysis in single-cell microscopy figures, Related to STAR Methods.

Figure	Label in figure	Strain	Sample size (n)
Figure 2D	NLS-NES-GFP	MO115	230
	Clb2-Citrine	MO251	49
	Cdc6-Citrine	MO256	24
Figure 2E-G, S2C	CDC6(WT)	MO248	232
	CDC6(WT) sic1	MO250	112
	CDC6(WT) cdh1	MO249	107
	cdc6(lxf)	MO253	104
	cdc6(lxf) sic1	MO254	113
	cdc6(lxf) cdh1	MO255	102
Figure 3C-D, S3A	Cdc6(WT)	MO235	183
	Cdc6(T39A S43A)	MO237	245
	Cdc6(T368A S372A)	MO258	406
	Cdc6(lxf)	MO268	275
	Cdc6(T368A S372A lxf)	MO184	361
Figure 3I	Cdc6(WT)	MO235	177
	Cdc6(WT) cdh1	MO302	186
	Cdc6(T368A S372A) cdh1	MO304	330
	Cdc6(T368A S372A lxf) cdh1	MO303	119
	Cdc6(T39A S43A T368A S372A lxf) cdh1	MO474	365
Figure 4A-C	CLB2(wt)	MO115	230
	clb2(hpm)	MO116	138
	clb2(hpm) + CLB2(wt)	MO155	118
	spo12	MO227	120
	spo12(lxf)	MO232	128
	spo12(+RxL)	MO281	121
Figure 5C	Swe1(wt)	MO343	94
	swe1	MO347	96
	swe1(lxf1)	MO360	101
	swe1(lxf23)	MO349	96
	swe1(T196S)	MO356	103
Figure 6D	wild type	MO340	34
	clb2(hpm)	MO341	26
	bni1(lxf)	MO342	36

Figure 6E	wild type	MO340	132
	clb2(hpm)	MO341	108
	bni1(lxf)	MO342	191
Figure S3D-E	Cdc6(wt)	MO235	177
	Cdc6(T39A S43A)	MO237	234
	Cdc6(T368A S372A)	MO258	280
	Cdc6(rxl2)	MO236	181
	Cdc6(T7S T23S)	MO238	215
Figure S3G	Clb2-Citrine	MO251	49
	Clb2-Citrine cdh1	MO261	55
